# Nuclear and chloroplast diversity and phenotypic distribution of rice (*Oryza sativa* L.) germplasm from the democratic people’s republic of Korea (DPRK; North Korea)

**DOI:** 10.1186/s12284-014-0007-4

**Published:** 2014-07-02

**Authors:** HyunJung Kim, Eung Gi Jeong, Sang-Nag Ahn, Jeffrey Doyle, Namrata Singh, Anthony J Greenberg, Yong Jae Won, Susan R McCouch

**Affiliations:** 1 Department of Plant Breeding and Genetics, Cornell University, Ithaca 14853, NY, USA; 2 Rural Development Administration (RDA), Suwon 441-707, Republic of Korea; 3 Department of Crop Science, Chungnam National University, Daejeon 305-764, Republic of Korea; 4 Department of Plant Biology, Cornell University, Ithaca 14853, NY, USA

**Keywords:** DPRK, Germplasm, Diversity, temperate japonica, indica

## Abstract

**Background:**

Rice accounts for 43% of staple food production in the Democratic People’s Republic of Korea (DPRK). The most widely planted rice varieties were developed from a limited number of ancestral lines that were repeatedly used as parents in breeding programs. However, detailed pedigrees are not publicly available and little is known about the genetic, phenotypic, and geographical variation of DPRK varieties.

**Results:**

We evaluated 80 *O. sativa* accessions from the DPRK, consisting of 67 improved varieties and 13 landraces. Based on nuclear SSR analysis, we divide the varieties into two genetic groups: *Group 1* corresponds to the *temperate japonica* subpopulation and represents 78.75% of the accessions, while *Group 2* shares recent ancestry with *indica* varieties. Interestingly, members of *Group 1* are less diverse than *Group 2* at the nuclear level, but are more diverse at the chloroplast level. All *Group 2* varieties share a single *Japonica* maternal-haplotype, while *Group 1* varieties trace maternal ancestry to both *Japonica* and *Indica*. Phenotypically, members of *Group 1* have shorter grains than *Group 2,* and varieties from breeding programs have thicker and wider grains than landraces. Improved varieties in *Group* 1 also show similar and/or better levels of cold tolerance for most traits, except for *spikelet number per panicle*. Finally, geographic analysis demonstrates that the majority of genetic variation is located within regions that have the most intensive rice cultivation, including the Western territories near the capital city Pyungyang. This is consistent with the conscious and highly centralized role of human selection in determining local dispersion patterns of rice in the DPRK.

**Conclusions:**

Diversity studies of DPRK rice germplasm revealed two genetic groups. The most widely planted group has a narrow genetic base and would benefit from the introduction of new genetic variation from cold tolerant landraces, wild accessions, and/or cultivated gene pools to enhance yield potential and performance.

## Background

Rice plays a critical role in food security in Asian countries. Despite its importance, achieving a regular, stable supply of rice in the developing world is still difficult, due to inherent inelasticity of markets, domestic politics, and lack of genetic and scientific resources within some developing countries (Wailes and Chavez [2012]; ERS-USDA [2012]). Unlike other major staple cereals, humans consume most rice directly as a whole grain, and only a small percentage (~7%) of rice is exported across sociopolitical borders (FAO [2012]). This consumption pattern has profound consequences for the eco-regional forms of adaptation found within rice cultivars and drives the selection of locally preferred grain quality characteristics (Fitzgerald et al. [2009]). Within this manuscript, we focus on genetic and phenotypic variation found in rice from the Democratic People’s Republic of Korea (DPRK: North Korea). The DPRK’s complete isolation has preserved the local genetic variability to an extent that is not possible in countries with more open borders, for people, as well as for grains. Knowledge of the genetic variability within rice from the DPRK could potentially benefit the serious food security issues that many of its citizens face.

The DPRK lies within the temperate zone and has depended on rice as a staple food for centuries. Based on archaeological records, the first rice cultivation on the Korean peninsula dates back to the late Neolithic era, approximately 3,000 B.C., while evidence from various sites across the peninsula suggest that irrigated paddy culture flourished during the Bronze Age about 1,300-300 B.C. (Ahn [2010]). Archeo-botanical evidence suggests that rice arrived in Korea in its ancient domesticated form, having short and round grains that resemble modern forms of cultivated *temperate japonica* rice (Shim [1991]; Kim et al. [2013]). This view is supported by the fact that there is no wild ancestral rice, *O. rufipogon* or *O. nivara,* found on the Korean peninsula. Biological evidence from weedy rice and native varieties collected from 1905–1920 also supports the existence of *temperate japonica* rice as the major cultivated form in the region. There is little evidence to suggest that *indica* rice was introduced to Korea before 1920s (Heu et al. [1991]; Suh et al. [1992]; Kwon et al. [2000]). During the Green Revolution, scientists in the DPRK made some of the first *indica* × *temperate japonica* crosses using *indica* germplasm from the International Rice Research Institute (IRRI) (Dalrymple [1986]).

In the DPRK today, 16% of the total land area is cultivated, and rice is produced on cooperative farms run by the government owned Public Distribution System (PDS). Rice accounts for 43% of staple food production in North Korea, while maize, barley, wheat, soybean and potato make up the rest of the food staples produced by PDS (WFP et al. [2011]; UN [2011]). Rice production is concentrated in the heavily populated southwestern provinces, including the capital city Pyungyang, whereas the rural northeastern regions mostly produce maize and other crops. The majority of the crops produced in the DPRK are consumed locally.

In general, the growth conditions for rice in DPRK are not favorable. Short duration of the growing season, erratic distribution of rainfall, and restricted availability of fertilizer all have adverse effects on yield. However, cold damage is the most serious and common problem, and cold tolerance is an essential trait for rice varieties in DPRK (KREI Quarterly Agricultural Trends in North Korea, (http://www.krei.re.kr/web/www/26, in Korean).

Most of the cultivated rice varieties in the DPRK today were developed 25–30 years ago using traditional breeding methods and have very similar genetic backgrounds, due to the repeated use of a limited number of ancestral lines as parents in breeding programs. Reports from 1998 suggest that three varieties, Pyeongyang 15, Pyeongyang 18 and Pyeongyang 21, were cultivated on as much as 80% of rice paddies in DPRK (Kim et al. [1998]); however, little is known about the genetic or phenotypic variation of these varieties, nor about the identity or pedigrees of other more current varieties.

The only information currently available on genetic diversity within DPRK rice varieties comes from an Amplified Fragment Length Polymorphism (AFLP) markers study (Cho et al. [2002]). South Korean researchers at the National Institute of Crop Science (NICS) in the Rural Development Administration (RDA) conducted several other studies looking at phenotypic variation associated with yield components and cold tolerance (Kim et al. [1996]; Noh et al. [1997]; Jeong et al. [2000]).

Building on previous work, this study analyzes genotypic and phenotypic variation for 80 *O. sativa* accessions collected in DPRK by South Korean researchers during 1970s-1990s. We used SSR and sequence-derived markers to examine the population structure of these 80 varieties and evaluate genetic diversity in both the nuclear and chloroplast genomes. We also examined morphological variations in grain traits based on population structure. Together with our analysis of cold tolerance-related traits within DPRK improved-*temperate japonica* varieties, we hope our efforts will help guide future plant breeding efforts in the DPRK to develop more cold-tolerant rice varieties with improved yields.

## Results

### Population structure in DPRK germplasm

**Figure 1:**
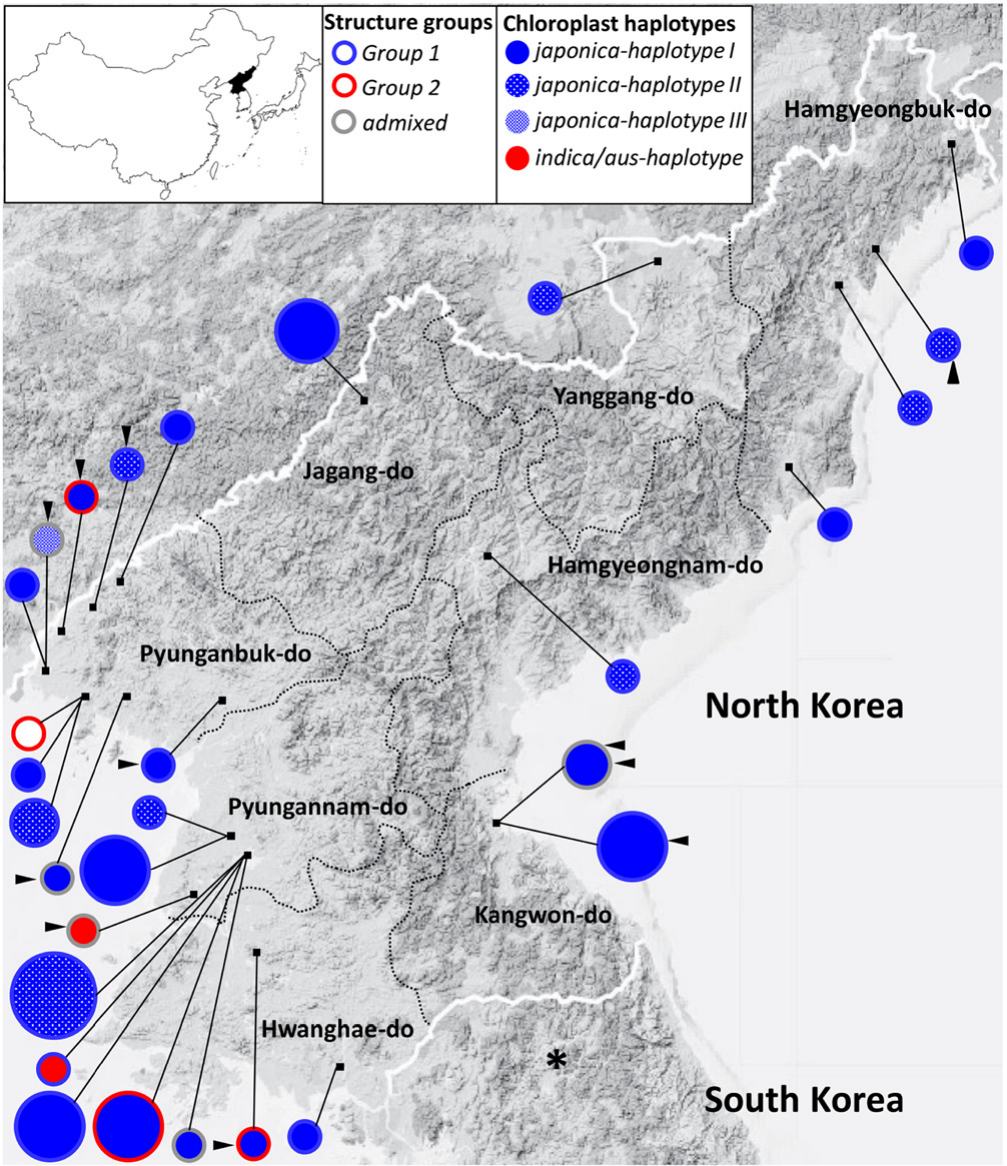
**Sampling loci on eight provinces and genetic information of Structure groups and chloroplast haplotypes of DPRK rice germplasm.** Circle size corresponds to number of samples. Outline of circle gives Structure information (Figure 2A) and circle is filled with chloroplast haplotypes (Figure 3). Landraces are designated as the closed triangle on the circle. Field phenotyping locality in South Korea is indicated with a star. Detail information of genetic codes is shown in Table S1.

**Figure 2:**
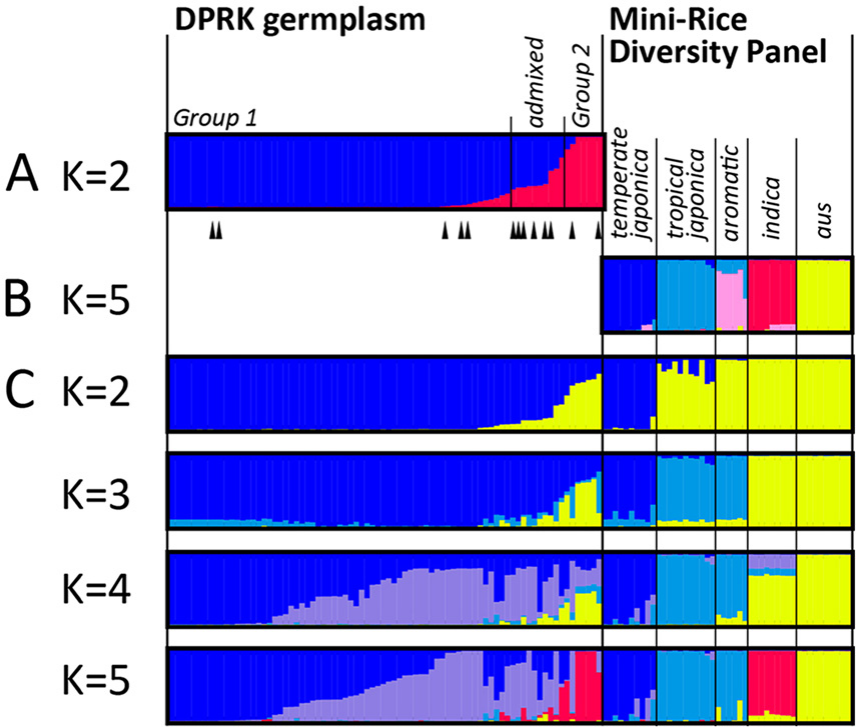
**STRUCTURE analysis. (A)** DPRK germplasm at K = 2. Landrace accessions are indicated with triangles below graph; **(B)** Mini-Rice Diversity Panel at K = 5; C. combined DPRK germplasm and Mini-Rice Diversity Panel at K = 2-5

When 80 DPRK accessions (Figure 1, Additional file 1: Table S1) were evaluated using population structure analysis method based on 51 nuclear SSR (nSSR) loci, best fit was observed when the population was divided into two groups, K = 2 (Additional file 2: Figure S1A). The majority of accessions (63) clustered within one subpopulation, which we refer to as *Group 1*, while 7 accessions were grouped into a second subpopulation, *Group 2* (Figure 2A). The remaining ten accessions, including 6 landrace varieties that share less than 80% ancestry with either group, were considered admixed (Falush et al. [2007]). Among the three known varieties widely cultivated in DPRK, Pyeongyang 15 belonged to *Group 1* while two other varieties, Pyeongyang 18 and Pyeongyang 21 belonged to *Group 2*. In landraces, 6 out of 13 were clarified as *admixed* group.

### Genetic relationship between DPRK germplasm and *O. sativa* diversity panel

Genotypic data from 40 nSSR loci were analyzed using STRUCTURE to determine the relationship between the two major DPRK subpopulation groups and the five subpopulations previously reported using the “Mini-Rice Diversity Panel” (Garris et al. [2005]; Zhao et al. [2010]; Huang et al. [2008]). As shown in Figure 2B (and Additional file 2: Figure S1B), the five subpopulations were clearly identified in the Mini-Rice Diversity Panel using these markers, namely, *temperate japonica*, *tropical japonica*, *aromatic*, *indica* and *aus*. When the DPRK accessions and the Mini-Rice Diversity Panel were analyzed together, the highest delta K value was K = 2 (Additional file 2: Figure S1C). At K = 2, the DPRK varieties of *Group 1* were composed of the *temperate japonica* subpopulation, while the DPRK varieties of *Group 2* were composed of the other four subpopulation groups and clustered into one group (Figure 2C). At K = 3, the *aromatic* and *tropical japonica* subpopulations formed a separate group. With K = 5, the *aus* group was separated from *indica*, and most of the *Group 2* accessions clustered with *indica.*

Results from analysis of genetic distances (Cavalli-Sforza and Edwards [1967]) and principal component analysis (PCA) were consistent with Structure analysis (Additional file 2: Figure S2).

### Chloroplast haplotype network

**Figure 3:**
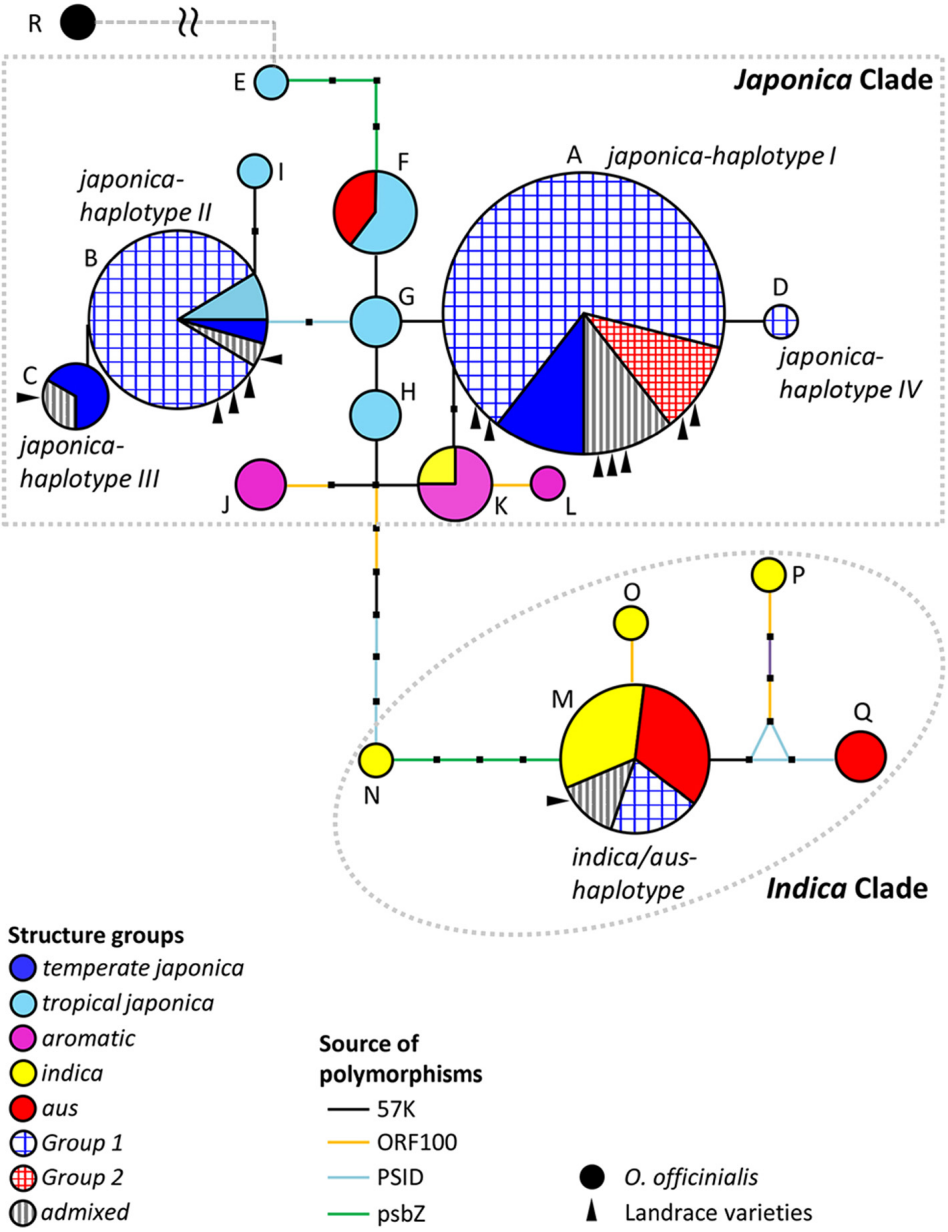
**Chloroplast haplotype network based on sequence information.** Circle size corresponds to no. of samples and circle is filled with Structure information. Landrace accessions are indicated with triangles. Bars indicate mutation events.

Five haplotypes were detected in DPRK rice germplasm based on sequence analysis of 4,131 bp of the chloroplast. When analyzed with Mini-Rice Diversity Panel, haplotypes were separated into two major clades, the *Japonica* clade and the *Indica* clade, by 7 mutational steps including the 69 bp indel in ORF100 (Figure 3 and Additional file 1: Table S1) (Kanno et al. [1993]). Haplotypes A-D (hereafter referred to as *japonica-haplotypes I-IV*) were located in the *Japonica* clade and haplotype M (hereafter referred to as *indica/aus-haplotype*) was found within the *Indica* clade. The most common haplotype, *japonica-haplotype I*, was shared with 65.4% of DPRK accessions and 66.7% of *temperate japonica* accessions from the Mini-Rice Diversity Panel. By contrast, the *japonica-haplotype IV* was rare, observed in a single improved variety (Nong 57). Three haplotype groups (*japonica-haplotypes I, II* and *indica/aus-haplotype*) represented the largest proportion of DPRK germplasm and contained both landraces and improved varieties. None of the DPRK haplotypes were shared with the *aromatic* subpopulation from the Mini-Rice Diversity Panel.

We identified 59 DPRK accessions belonging to *Group 1* and they retained *japonica-haplotypes I, II* or *IV*. By contrast, not a single accession had both *Indica* chloroplast and nuclear genomes: while all the *Group 2* varieties had *japonica-haplotype I,* the varieties harboring the *indica/aus-haplotype* belonged to *Group 1* or *admixed*-group. For instance, the glutinous accession, “Yukchal” of *Group 1* retained the *indica* haplotype. This result suggests that the 4 *Indica/Japonica* varieties in *Group 2* used *temperate japonica* as a maternal source.

The two main chloroplast clades were consistently separated in topology of the haplotype tree based on combined sequencing and length polymorphism analysis (Additional file 2: Figure S3).

### Genetic similarity within DPRK varieties based on nuclear and chloroplast SSRs

**Figure 4:**
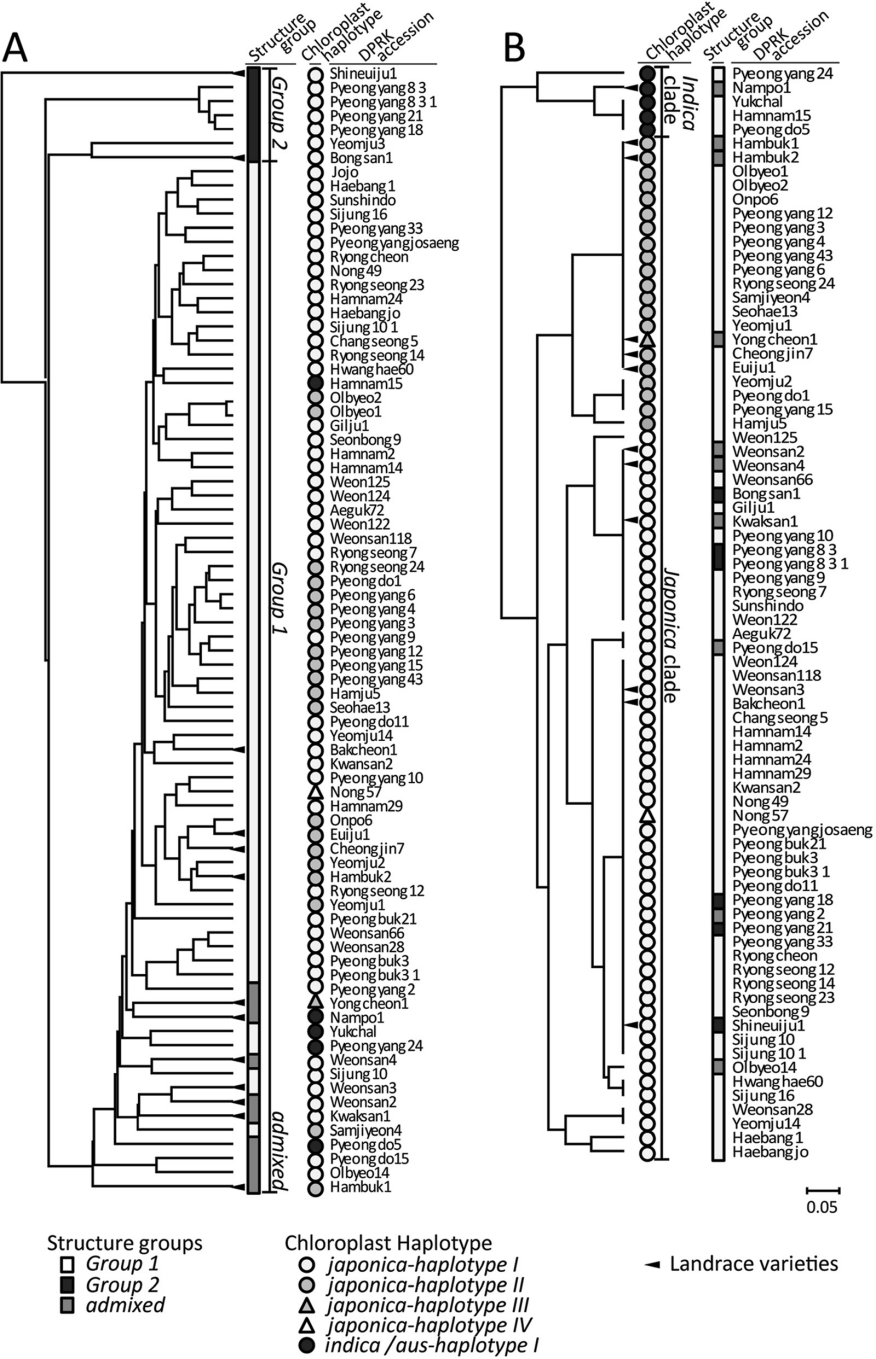
**Dendrogram based on genetic similarity within DPRK varieties using nSSRs (A) and cpSSRs (B).** The samples are also identified by sub-population code in Figure 2A and chloroplast haplotype in Figure 3.

The largest cluster was composed of 63 DPRK accessions from *Group 1* and admixed, as identified by STRUCTURE analysis based on 51 nSSRs (Figure 4A). The 7 DPRK varieties belonging to *Group 2* formed three separate clusters composed of only 1–4 individuals each. The most divergent of these clusters was represented by a single landrace from Shineuiju, followed by a group of four improved *Indica/Japonica* rice varieties named “Pyeongyang 8, 18, and 21”, a landrace called Bongsan1, and an improved variety, Yeomju 3. Based on pairwise similarity, the landrace from Shineuiju1 was most distant from the improved variety Yeomju1 (D = 0.821), while the two improved varieties, Olbyeo1 and Olbyeo2 were most closely related to each other (D = 0.018).

The analysis of genetic distance based on chloroplast SSRs (cpSSR) among DPRK accessions showed results consistent with the chloroplast haplotype network; the five DPRK accessions carrying the *indica*/*aus-haplotype* (as defined in Figure 3) were clearly separated from the accessions carrying the *japonica-haplotypes*. Accessions carrying *japonica-haplotypes I* and *IV* could also be distinguished from *japonica-haplotypes II* and *III* (Figure 4B).

### Genetic differentiation and diversity

*Group 2* showed greater diversity, measured by allelic richness (standardized measure of the average number of alleles per locus) and gene diversity (Table 1A) than *Group 1* in the nucleus. By contrast, *Group 1* demonstrated greater chloroplast diversity. The different level of genomic diversity measured in nuclear and chloroplast genomes can be attributed to their disparate method of inheritance and the genomic mutation rate. Unlike nuclear genomes, plastid genomes are maternally inherited and have a lower mutation rate than nuclear genomes. Moreover, Poaceae plants, including rice, are known to have a very low frequency of leakage of paternal plastomes (Corriveau and Coleman [1988]; Tang et al. [2004]; Azhagiri and Maliga [2007]). Taken together, we can conclude that a genetically narrow range of maternal *japonica* parents were used in crosses with *indica* to generate *Group 2* varieties.

**Table 1:** Genetic diversity and population differentiation

**A. Genetic diversity**									
Population		Nucleus			Chloroplast				
		No. of samples	Gene diversity	Allelic richness	No. of samples	Gene diversity	Allelic richness		
DPRK	*Group1*	63	0.304	2.007	62	0.134	1.466		
	*Group2*	8	0.441	2.240	6	0.046	1.083		
	*admixed*	10	0.454	2.418	10	0.255	1.674		
Mini-Rice Diversity Panel	*temperate japonica*	10	0.280	1.912	9	0.113	1.248		
	*tropical japonica*	11	0.385	2.312	11	0.152	1.459		
	*aromatic*	5	0.331	1.742	5	0.246	1.457		
	*indica*	9	0.522	2.722	9	0.293	1.734		
	*aus*	10	0.470	2.486	10	0.207	1.640		
**B. Pairwise Fst and Rst based on nSSRs**^**♩**^									
Model based group		*Group1*	*Group2*	*admixed*	*temperate japonica*	*tropical japonica*	*aromatic*	*indica*	*aus*
DPRK	*Group1*	0	0.15698*	0.02852	0.31672**	0.30472**	0.28058*	0.44312**	0.43371**
	*Group2*	0.44127**	0	0.06471	0.46329**	0.43001**	0.41775**	0.32082**	0.44695**
	*admixed*	0.13387**	0.27433**	0	0.41645**	0.41255**	0.3671**	0.42727**	0.49235**
Mini-Rice Diversity Panel	*temperate japonica*	0.17589**	0.50115**	0.24504**	0	0.36977**	0.39332**	0.55988**	0.51885**
	*tropical japonica*	0.42686**	0.42243**	0.36964**	0.44131**	0	0.37587**	0.50219**	0.44887**
	*aromatic*	0.56358**	0.55827**	0.49773**	0.62088**	0.39265**	0	0.61571**	0.52853**
	*indica*	0.59369**	0.33043**	0.41700**	0.57704**	0.50522**	0.55020**	0	0.39948**
	*aus*	0.60742**	0.43762**	0.47938**	0.60109**	0.49746**	0.49755**	0.35632**	0
**C. Pairwise Fst based on chloroplast haplotype**									
Model based group		*Group1*	*Group2*	*admixed*	*temperate japonica*	*tropical japonica*	*aromatic*	*indica*	*aus*
DPRK	*Group1*	0							
	*Group2*	0.12911	0						
	*admixed*	0.01667	0.15174	0					
Mini-Rice Diversity Panel	*temperate japonica*	0.02233	0.10224	−0.05469	0				
	*tropical japonica*	0.31411**	0.46612**	0.18716**	0.2543**	0			
	*aromatic*	0.41235**	0.63359**	0.26113**	0.34396**	0.14715*	0		
	*indica*	0.42547**	0.61186**	0.22359**	0.38889**	0.16664*	0.27747**	0	
	*aus*	0.42271**	0.60000**	0.21509**	0.38722**	0.21908**	0.24837**	0	0

**♩** Fst below diagonal and Rst above diagonal.

* P-values <0.05.

** P-values <0.01.

Further analysis employing Fst and Rst statistics indicated significant differences between the two DPRK varietal groups (Fst = 0.44127 at P = 0.000, Rst = 0.15698 at P ≤ 0.05) as well as differences between the other five subpopulations in the Mini-Rice Diversity Panel (Table 1B). Using Fst values, *Group 1* was genetically most similar to the *temperate japonica* subpopulation, while *Group 2* was most similar to *indica*.

When chloroplast haplotype data were used to compare groups based on pairwise Fst, the two DPRK groups did not significantly differ from one another (Table 1C) or from the *temperate japonica* group in the Mini-Rice Diversity Panel. This suggests that breeding in both groups involved *japonica* female lines and is consistent with the disparity observed when nuclear or chloroplast marker data is used to cluster the DPRK varieties.

The *admixed* group showed greater allelic richness and higher gene diversity than other groups and was genetically closer to *Group 1* than *Group 2* based on both nuclear and chloroplast. These results are consistent with analysis of Structure and genetic distance shown in Figure 2A and Figure 4A.

We compared Fst and Rst statistics to understand causes of population differentiation *i.e.* drift vs. mutation or high vs. low gene flow. According to the Stepwise Mutation Model (Balloux and Goudet [2002]; Hardy et al. [2003]), Rst is larger than Fst for microsatellites mutations. Yet pairwise comparisons between *Group 1* and *Group 2* based on nSSRs showed that Fst values were much greater than Rst values. The same pattern was observed when each of DPRK groups and other subpopulations in the Mini-Rice Diversity Panel were compared. These data suggest that gene flow or drift may be responsible for the differentiation between almost all of the subpopulations. On the other hand, a comparison between *Group 1* and *temperate japonica* (Mini-Rice Diversity Panel) indicated that Rst values were greater than Fst values. We can therefore attribute differentiation between *Group 1* and *temperate japonica* varieties originating in South Korea, Japan and China to mutation, observed as an accumulation of step-wise mutations over time (Balloux and Goudet [2002]).

When landraces were compared to improved varieties using analysis of molecular variance (AMOVA), only 8.17% (P value < 0.0001) of the total genetic variance was due to differentiation between landraces and improved varieties.

### Comparison of genetic and geographic distance

Fifty DPRK individuals had city-level collection information, as well as the genetic distance estimates based on data from 51 nSSRs. For these individuals, no significant correlation was observed between genetic and geographical distance based on a Mantel test. Rather, a wide range of genetic variability (from 0.035 to 0.636) was observed among accessions collected from the same city.

Based on pairwise Fst analysis among 8 provinces, 3 major rice provinces in the western coastal areas, Pyungannam-do, Pyunganbuk-do and Hwanghae-do were not genetically different, while three provinces in the eastern mountainous area, Hamgyeongnbuk-do, Hamgyeongnnam-do, and Kangwon-do showed genetic differences (Figure 1 and Additional file 1: Table S2).

### Phenotypic variations

**Figure 5:**
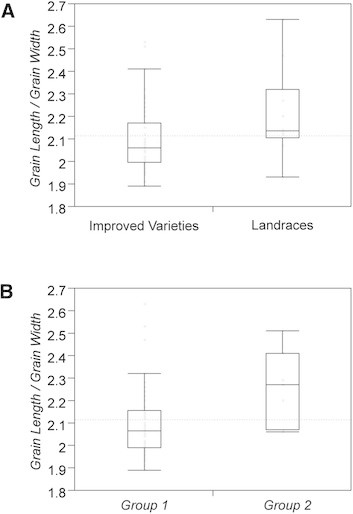
**Grain length/width ratio (A) between improved varieties and landrace varieties and (B) between** ***Group 1*** **and** ***Group 2*.**

Differences in grain shape were observed between the two varietal groups, *Group 1* and *Group 2,* and between improved varieties and landraces. *Group 1* accessions were significantly shorter grain (*GL*) than those of *Group 2* and DPRK improved varieties had significantly wider (*GW*) and thicker (*GT*) grains than the landrace varieties (Additional file 2: Figures S4). As a result, both *Group 2* and landrace varieties had a higher *grain length*/*width ratio* (*GLWR*) than *Group 1* and improved varieties, respectively (Figure 5). Shorter *GL* in *Group 1* compared to *Group 2* varieties was consistent with observed differences in grain shape reported between *temperate japonica* and *indica,* or *Indica/Japonica* varieties (RDA and NARI [2012]; Takano-Kai et al. [2009]). Awns were observed in 15% of DPRK materials, while no significant variation in awns were found between two genetic groups and between landraces and improved varieties.

A comparison of cold tolerance between *Group 1* and *Group 2* accessions and between landraces and improved varieties was not possible due to a lack of phenotypic data for *Group 2* and landrace materials. Having said this, we estimated cold tolerance response for the improved-*Group 1* varieties based on differences between cold-treated and reference conditions, as summarized in Additional file 2: Figure S5.

Low temperature-treatment inhibited plant growth of *Group 1* accessions; plants irrigated with cold water (17°C) had significantly greater *days to heading (DTH)* (av. difference = 12.75 days, *t* = 40.584***), smaller *culm length* (*CL)* (av. difference = 18.212 cm, *t* = −21.512***), fewer panicle numbers (*PN*) (av. difference = 0.827, *t* = −2.311*), fewer *spikelet number per panicle* (*SPP)* (av. difference = 52.769 spikelets, *t* = −13.713***) and lower *percentage of seed set* (*PSS)* (av. difference = 32.596%, *t* = −14.446***) than control plants based on a paired t-test. Additionally, this abiotic stress affected *leaf discoloration* (*LDS* or yellowing) (av. = 3.077 ± 1.355 with 1 as complete green) and incomplete *panicle exertion* (*PE*) (av = 4.423 ± 1.819 with 1 as complete exertion).

In correlation analysis, we observed significant phenotypic relations under the cold stress (Additional file 1: Table S4); we scored 1 out of 9 as the most tolerant in cold tolerance related traits such as *PE*, *phenotype acceptability at tillering* (*PAT)*, and *phenotype acceptability at maturity* (*PAM*). Strong tolerant responses were commonly related to earlier *DTH*, shorter *CL*, higher *PSS* and less *reduction ratio of PSS* (*R_PSS)*. Another cold related trait, *LDS*, yellowing on the leaf was correlated with delayed days to heading (*D_DTH)* and increased *reduction ratio of SPP* (*R_SPP*).

In addition to the genetic traits discussed above, we also note specific phenotypes of notable breeds of rice we studied. Among three varieties widely grown in DPRK, Pyeongyang 15 of *Group 1* had longer grain (7.99 mm) than most of *Group 1* varieties (av. = 7.54 mm) and higher *PSS* in cold water (77%, compared to av. = 53.27%). Pyeongyang 18 of *Group 2* had short *CL* than the rest of its members (76 cm, compared to av. = 88.5 cm). Pyeongyang 21 of *Group 2* was sensitive to cold temperature; heading was delayed by 17.35 days (compared to av. = 12.96 days), while 58.8% of *SPP* was decreased (compared to av. = 37.90%) and 43.5% of *PSS* was reduced (compared to av. = 38.31% reduced). Yellowness in the leaf was also increased as a result of chilling (score as 5, compared to av. = 3.08).

## Discussion

### Population structure and genetic diversity

Two sub-groups were identified within DPRK rice germplasm using nuclear microsatellites. A majority (78.75%) were identified as *Group 1* varieties and clustered with *temperate japonica* control varieties, while *Group 2* varieties showed evidence of *indica* ancestry. *Group 2* had greater allelic diversity at the nuclear level, and could be sub-partitioned into three groups using UPGMA. One of the subgroups included the *Indica*/*Japonica* rice variety (Pyeongyang 18) that shared recent ancestry with semi-dwarf *indica* varieties, and corresponded to the second group, Tongil-type rice varieties, identified in Cho et al. ([2002]) (Note: Tongil-type rice varieties are also *Indica/Japonica* varieties, developed from South Korea during Green Revolution in 1970s). Two other subgroups within *Group 2* were resolved at K = 4 (when the DPRK germplasm was analyzed alone) or at K = 5 (when the DPRK plus the Mini-Rice Diversity Panel were analyzed together). Both of these sub-groups were classified as admixed; one characterized by *temperate japonica* and *indica* ancestry, and the other by various *temperate japonica* lineages. The three admixed subgroups within *Group 2* were not well differentiated based on Structure analysis due to comparatively small number of *Group 2* to *Group 1*.

The population structure inferred from analysis of nSSRs was largely, but not entirely, consistent with estimates of ancestry based on the maternally inherited, minimally recombinant chloroplast genome. A majority (95.16%) of *Group 1* accessions carried the *japonica-haplotypes* in the chloroplast, while 4.84% carried the *indica/aus* chloroplast. None of *Group 2* carried the *indica/aus* chloroplast haplotype; rather they all carried the *japonica-haplotype I*, suggesting that the maternal parent used to generate the *Indica/Japonica* varieties was a *temperate japonica*. Further, the major chloroplast-defined genetic group, *japonica-haplotype I,* was more genetically diverse than the *indica/aus-haplotype* group, in sharp contrast to the diversity of the nuclear genome-defined groups.

### Population differentiation

The Fst statistic relies solely on measureable differences in allele identity, while the Rst statistic incorporates both allele identity/nonidentity and allele size differences under the stepwise mutation model (Hardy et al. [2003]). While Fst is generally more effective in estimating population divergence due to high gene flow, Rst is useful for estimating population differentiation under low gene flow. Comparing Fst and Rst values computed on the same data can provide valuable insights into the main causes of population differentiation. In this case, we used the comparison between Fst and Rst to evaluate the relative importance of gene flow and drift vs. step-wise mutation. The comparison revealed interesting phylogeographic patterns, suggesting that a small pool of founder lines from *temperate japonica* may have come to the Korean Peninsula. Subsequent inbreeding coupled with an accumulation of stepwise mutations occurred over a long enough period of time to restore rare alleles and give rise to new ones, differentiating the derived *Group 1* population from its *temperate japonica* source population. However, a majority of the DPRK rice accessions in this study represented improved varieties developed over the last 60 years, which were genetically very narrow in both genomes. These data suggest that strong selection was applied to develop DPRK rice varieties and that a few varieties were often reused as parental lines in the breeding programs. Despite a lack of historical information about DPRK rice genealogy, it is widely acknowledged that DPRK rice varieties have been strongly selected to improve adaptation, yield and quality (Kim et al. [1998]).

### Spatial analysis

No significant isolation by distance was observed when pairwise genetic distance was regressed against the pairwise geographic distance among varieties. Rather, a majority of the genetic variation was found clustered in a single province, Pyungannam-do, where the capital city Pyungyang is located. This province and others located in the Western territories of the DPRK (Pyunganbuk-do and Hwanghae-do) contain 77.6% of irrigated rice fields in the country (Shin et al. [2000]). Thus, the region of most intensive rice cultivation contains the highest concentration of rice genetic diversity, which is consistent with a conscious and highly centralized role of human selection in determining local dispersion patterns of rice in the DPRK. Rice germplasm is typically collected and managed in the main breeding centers and subsequently disseminated to more distant provinces.

### Landrace varieties

The 13 landraces included in this study were collected mainly from the area around the large rice plains in Pyunganbuk-do and Kangwon-do (Figure 1). Evidence of cultivation of landraces directly in farmers’ fields is hard to come by and the resolution of this study is insufficient to indicate whether the landraces have been actively used to improve rice cultivars in recent breeding efforts in the DPRK. Additionally, most of landraces were awnless and belonged to *Group 1* (n = 5) or *admixed* (n = 6). This information suggests that most of the landraces in this study were recently derived from cultivated rice or intraspecific crosses between *Group 1* and *Group 2*.

### Historical model of genetic bottleneck to DPRK

Historical backgrounds may better explain genetic bottleneck of DPRK varieties to *Group* 1, *temperate japonica*. As previously mentioned, it is believed that a genetically bottlenecked form of rice arrived in the Korean peninsula from an era of early rice cultivation, as a domesticate that was already adapted to the temperate zone. Once on the peninsula, early breeder-farmers imposed selection for the preferred grain quality for consumption. Notably, they selected for non-aromatic grains with a chewy texture for the main meal, and for sticky, glutinous rice as the source for cakes and desserts for festivals and other special occasions, consistent with the historical record (Ilyeon [1281]). While there is little historical evidence of another ecotype of rice similar to *indica*, it would not have been used widely in the DPRK due to its unsuitability to cool temperate areas.

Before the Korean War (1950–1953), only 25% of total rice production was located in the northern areas of the Korean Peninsula (current DPRK), where 80% of the land is mountainous terrain (UNEP [2003]). Rice was largely produced in the southern areas (current South Korea; Republic of Korea), which accounted for the rest of the production. At that time, the Agricultural Demonstration Station of the Great Korean Empire (founded in 1906, current RDA, South Korea) served as the lead institution for the collection of rice germplasm, development and distribution of rice varieties for both northern and southern areas (http://www.rda.go.kr). After the Korean War, the practices of DPRK breeders have been closed to the scientific community, so we have little knowledge of the resources and methodologies used for crop improvement. However, this study and related studies suggest that the DPRK is working with a narrow base of genetic diversity in terms of rice genetic resources which may constrain its potential for genetic gain in its breeding programs (Moon and Ahn [1998]; Cho et al. [2002]).

During the green revolution in the 1970s, the DPRK introduced allelic variation from high-yielding and blast resistant *indica* varieties through *Indica/Japonica* hybridization, giving rise to the Pyeongyang accessions of *Group 2* in this study. However, those lines from interspecific crosses were not adopted widely because of their taste and their sensitivity to cold stress as compared to pure *temperate japonica* (Moon and Ahn [1998]). It is generally acknowledged that DPRK breeders have imposed strong selection for high-yield and high-density cultivation above other agronomic traits. As a result, many varieties are susceptible to diseases, insects and lodging (Kim et al. [1998]). For example, 16% of DPRK improved varieties in this study had a white awn, which is generally selected against in modern breeding programs (Sweeney and McCouch [2007]), but was a common seed character in 80% of native or landrace Korean varieties collected during 1911–1923 (Choi [2005]).

### Agronomic traits of DPRK germplasm

That significantly longer grains were found in *Group 2* varieties, as compared to *Group 1,* is consistent with observed differences between *temperate japonica* and *indica* or *indica*/*japonica* varieties (RDA and NARI [2012]; Takano-Kai et al. [2009]).

Cold water treatment during the reproductive stage induced a strong phenotypic response in improved varieties of *Group 1*. The responses were measured using various indicators, which we list here: shortened *culm length* (*CL*), delayed *days to heading* (*DTH*), and great *reduction ratio of spikelet number per panicle* (*R_SPP*) and *percentage of seed set* (*PSS).* The yield-related traits, *SPP* and *PSS* were also dramatically affected by cold stress. During the reproductive stage, cold stress negatively affects pollen development and generates high levels of sterility that lead to severe yield losses (Imin et al. [2006]; Suh et al. [2010]; Ishii et al. [2011]). Panicle number (*PN*) was not significantly reduced by the cold-treatment, however, *PN* is determined during the vegetative, rather than reproductive stage (Lee [2001]). We also note that the level of individual cold tolerance of improved varieties of *Group 1* did not have a strong regional dependence. Varieties from northern or southern areas, and from the main rice fields in western area or from eastern mountainous areas show similar cold tolerance levels.

We compared DPRK *temperate japonica* varieties (improved varieties of *Group 1*) with other *temperate japonica* varieties evaluated in a previous cold tolerance study in Chunchoen Substation, which used the same phenotypic methodology under the same conditions for both control and cold-treated sample (Suh et al. [2013]; Jeong et al. [2000]). DPRK varieties showed greater cold tolerance based on *PSS* and *phenotype acceptability at tillering* (*PAT)* than other *temperate japonica* varieties, but inferior cold tolerance in *SPP*. Other traits, such as *leaf discoloration* (*LDS)*, *difference in DTH* (*D_DTH*), *reduction ratio of CL* (*R_CL*), and *panicle neck exertion* (*PE*) were similar across the range of *temperate japonica* varieties. Consistently, the *SPP* of DPRK varieties treated with cold irrigation water was significantly lower than that of South Korean *temperate japonica* varieties used as donors of cold tolerance in breeding programs, as described in Kwon et al. [2002]. For example, among three major varieties, both Pyeongyang 15 and 21 lost more than 50% of *SPP* under cold environments. This suggests that improvement of *SPP* is essential for future breeding programs. It also suggests that improving *SPP* of DPRK varieties using some of the highly cold-tolerant South Korean donors in the DPRK breeding program would be possible.

Jeong et al. ([2000]) established threshold levels for six traits to define cold tolerance: *PSS* (>60%), *LDS* (1–3), *PAT* (1–3), *phenotype acceptability at maturity (PAM)* (1–3) under cold treatment and *D_DTH* (<10 days), and *R_CL* (<25) when comparing across treatments. We observed significant reductions of all of these traits for most of the varieties in our study (*PSS* (>60%), *LDS* (1–3), *PAT* (1–3), and *PAM* (1–3) under the cold condition, and *D_DTH* (<10 days), *R_CL* (<25%) but three *Group 1* varieties (Yeomju 1, Changseong 5, and Pyeongbuk 3_1) were less affected by the 17°C cold-water treatment than others. We recommend the use of these varieties by farmers in regions where reproductive-stage cold tolerance is required. These varieties may also be considered useful donors for further breeding of cold-tolerant varieties for the DPRK.

## Conclusions

A historical and geographic understanding of DPRK rice genetic resources reveals valuable insights for future rice improvement. Currently, the *Group 1* materials have the appropriate adaptation and quality characteristics for use as founder lines for crop improvement in the DPRK. However, to maximize grain yield, emphasis must be placed on the ability to sustain or increase yield under cold conditions and in the presence of other local forms of biotic and abiotic stress (Shin et al. [2000]; Jeong et al. [2000]; Kwon [2012]). The most effective way to achieve these goals is to introduce new genetic variation from landraces, wild accessions and/or elite, cultivated gene pools into the breeding programs in the DPRK.

## Methods

### Plant materials

A total of 127 rice accessions were analyzed in this study (Additional file 1: Table S1). This collection consisted of 80 DPRK varieties (67 improved varieties and 13 landraces), 46 diverse *O. sativa* varieties selected to represent the five subpopulations of *O. sativa* based on a previous study (Zhao et al., [2010], referred to as the “Mini-Rice Diversity Panel” panel) (10 *aus*, 9 *indica*, 6 *aromatic*, 11 *tropical japonica*, and 10 *temperate japonica*) and 1 accession of *O. officinalis* (IRGC 105220) as an out-group. The “Mini-Rice Diversity Panel” was used as a control to identify previously defined *O. sativa* subpopulation groups. The 80 DPRK varieties were collected in the DPRK by South Korean researchers during the 1970s-1990s, and included one glutinous accession, “Yukchal”. Information about the location of the collections was recorded at the city level for some varieties and the provincial level for others. Seeds of the 80 DPRK varieties, and two elite cultivars from South Korea, *Ilpum* and *Hwayeong,* are available through the National Agrobiodiversity Center (ARC in RDA; http://www.naas.go.kr) for research purposes only (with an MTA). Seeds of the “Mini-Rice Diversity Panel” are available through the Genetic Stock Center *Oryza* (http://www.ars.usda.gov/Main/Docs.htm?docid=23562).

Though the same language is spoken in North and South Korea, when translating the Korean language into English, the DPRK uses a different English spelling to represent Korean phonemes than does South Korea. In this paper, the South Korean tradition is followed for the spelling of geographical names.

DNA extraction was performed based on Dellaporta et al. ([1983]). PCR was performed based on Garris et al. ([2005]).

### Genotyping using nuclear and chloroplast markers

A total of 51 nSSR were selected from the larger pool of nSSRs available for rice based on the fact that they were well distributed throughout the genome, amplified well in multiplex arrays in both wild and cultivated germplasm, and were optimized for use in diversity analysis (Additional file 1: Table S3) (Garris et al. [2005]; Edwards [2005]).

Fluorescent-labeled nSSR primers were used for single-marker PCR and the amplified nSSRs were then multiplexed and sized on an ABI Prism 3730xl DNA analyzer (Applied Biosystems) using capillary electrophoresis at the Cornell Life Sciences Core Laboratories Center (CLC). Allele sizes were analyzed using the Peak Scanner v1.0 (Applied Biosystems) and GeneMapper v4.0 (Applied Biosystems).

The five chloroplast microsatellites of rice were first described by Ishii and McCouch ([2000]). They were assayed for polymorphism on 6% silver stained PAGE (Polyacrylamide Gel Electrophoresis) gels (Panaud et al. [1996]). In addition, five sequence-based chloroplast markers were also PCR-amplified and purified for direct sequencing from reverse and/or forward with an ABI Prism 3700/3100 DNA analyzer at the CLC. Sequences were aligned, called, and edited through CodonCode Aligner v3.5 (CodonCode Corporation). These markers were chosen from previous studies and showed polymorphism among AA genome accessions of rice such as *O. rufipogon*/*nivara* complex and/or *O. sativa*) (Ishii and McCouch [2000]; Kanno et al. [1993]; Kawakami et al. [2007]; Masood et al. [2004]; Takahashi et al. [2008]; Nakamura et al. [1998]). Aligned sequence data was blasted to Nipponbare (NC 001320, total length = 134,525 bp) in Genbank and physical positions were assigned. Sequence variations consisting of single and di-nucleotide polymorphisms (SNPs) and indels were identified using Tassel v3 (Bradbury et al. [2007]).

Fifty one nSSRs were used for population structure analysis and to build a dendrogram based on the 80 DPRK accessions and the two South Korean elite varieties. Previous work had utilized 40 of the 51 nSSRs for population structure analysis and to build phylogenetic trees based on a panel of 46 diverse *O. sativa* varieties and one accession of *O. officinalis* (Chung and Tyagi, Cornell University, personal comm.).

Haplotype network analysis was based on data from five sequence-based chloroplast markers and adding five length based cpSSR markers on all 127 accessions.

### Data analysis

For model-based cluster analysis, Structure 2.3.3 (Pritchard et al. [2000]) was used using 10 replicated runs each for populations K = 1-10, with run length = 500,000 iterations, followed by 100,000 burn-in length. An admixture model (Falush et al. [2007]) was used without prior information based on all accessions in this study. To determine the best K value, previous information about *O. sativa* population structure was used in combination with the Evanno method (Evanno et al. [2005]), implemented in Structure Harvester (Earl and VonHoldt [2012]). Using Clumpp, collated results were used to generate a mean and close match of permuted matrices across all replicates under the same cluster (Jakobsson and Rosenberg [2007]) and these results were visualized in distruct (Rosenberg [2004]). Analysis based on Structure and Clumpp programs were completed as multiple tasks through Computational Biology Service Unit (CBSU) in Cornell University. Samples that showed less than 80% shared ancestry with single population “K” were clustered as admixed. The multivariate method, PCA was performed using adegenet package v1.3-9.2 in R v3.0.2 (Jombart [2008]).

Genetic distance was estimated based on the chord distance of Cavalli-Sforza and Edwards ([1967]) $(\mathrm{Dc}=\frac{2}{\mathrm{\pi m}}\sum_{j=1}^{m}\sqrt{2(1-\sum_{i=1}^{\mathrm{aj}}\sqrt{\mathrm{pijqij})}}$, where *p*_*ij*_ and *q*_*ij*_ are the frequencies from the *i*th allele at the *j*th locus in two different populations). The *a*_*j*_ is the number of alleles at the *j*th locus while *m* is the total number of loci studied). An UPGMA dendrogram, the useful method for narrow genetic distanced accessions, was constructed to determine the genetic relationships among DPRK accessions based on dissimilarity matrix using PowerMarker V3.23 and visualized in Mega5 (Sneath and Sokal [1973]; Tamura et al. [2011]).

A parsimony haplotype network was built using TCS V1.21 (Clement et al. [2000]). Every sequence variation was appointed as a single polymorphism supposed as a single evolutionary event independently as the same weight. Gaps were treated as 5^th^ state (Simmons and Ochoterena [2000]). Three DPRK varieties, *Yeomju3*, *Jojo* and *Ilpum,* were excluded due to missing data. One haplotype network was built using chloroplast sequence-based polymorphisms and a second haplotype network was developed using SSR length polymorphisms in addition to sequence-based polymorphisms, where the SSRs were coded as length of PCR amplicon (Doyle et al. [1998]; Hale et al. [2004]).

Pairwise Fst and Rst statistics were used to compare population genetic models explaining divergence among subpopulations (step-wise mutation vs geneflow or drift) based on nuclear microsatellite markers (amplicon length) (Slatkin [1995]; Hardy et al. [2003]) using Arlequin v3.5 (Excoffier and Lischer [2010]). Pairwise Fst statistics were also calculated based on chloroplast haplotypes built from sequence-based markers. AMOVA was used to compare improved varieties and landraces by Arlequin v3.5.

Genetic diversity within sub-populations was calculated using Fstat V2.9.3 (Goudet [1995]) as Nei’s gene diversity and allelic richness (Nei [1987]). Isolation by distance was estimated at city level in km, converted from longitude and latitude of each regional site, using Mantel’s test with 10,000 randomization implemented by Isolation By Distance Web Service (IBDWS) V3.23 (Jensen et al. [2005]).

Estimates of genetic diversity were calculated using both nuclear and chloroplast genetic data. The number of alleles per locus, major allele frequencies, and gene diversity $\left({\hat{D}}_{l}=\left(1-{{\sum_{\mathrm{ju}=1}^{k}\tilde{p}}_{\mathrm{lu}}}^{2}\right)/(1-\frac{1+f}{n}\right)$ where *f* is the inbreeding coefficient, *n* is a number of individuals, *P*_*lu*_ is the population frequency of the *l th* allele at *u* locus) and polymorphism information content (PIC) $\left(P\hat{I}C_{l}=1-{{\sum_{u=1}^{k}\tilde{p}}_{\mathrm{lu}}}^{2}-{{\tilde{p}}_{\mathrm{lu}}}^{2}{{\tilde{p}}_{\mathrm{lv}}}^{2}{\hat{\sum }}_{u=1}^{k-1}\sum_{v+1}^{k}2{\hat{p}}_{2_{\mathrm{lu}}}{\hat{p}}_{2_{\mathrm{lv}}}\right)$ (Botstein et al. [1980]) were calculated on a per-locus basis using PowerMarker V3.23 (Liu and Muse [2005]).

### Agronomic traits of DPRK accessions

Cold tolerance on 57 of the DPRK improved rice accessions was evaluated by comparing trait performance in cold water-irrigated plots (treatment) to trait performance in control plots at the Chuncheon sub-station of NICS, South Korea during the summer of 2001. The following traits were evaluated in cold irrigated plots: *DTH*, *CL*, *PN*, *SPP*, *PSS*, *LDS*, and *PE*, *PAT*, and *PAM*, while the differences between treatment and control were labeled as *D_DTH*, *difference in PN* (*D_PN*), *R_CL*, *R_SPP*, and *R_PSS*.

Thirty day-old plants, seeded on April 25, 2001 and irrigated with 24°C water, were placed into control plots as well as cold tolerance-screening plots on May 25, 2001. Replicates consisted of 25 plants in a single row, and the experimental design consisted of a completely randomized block design with 2 replications. Row spacing between plants was 15 cm; column spacing was 25 cm. Plants for cold tolerance screening were irrigated with 24°C water until the tillering stage (30 days after transplanting) and then treated with 17°C cold-water until maturity. A water temperature of 17C was determined to be the critical temperature required to evaluate cold injury (Lee [2001]). As a control, a different set of thirty day-old seedlings was irrigated with 24°C water after transplanting until maturity.

Fully mature, dry seeds were used to evaluate five seed traits: *GL*, *GW*, *GT*, *GLWR*, and awns. For those phenotype, 10 randomly selected seeds from each of the 60 DPRK accessions for which seed was available were photographed and measured using ImageJ, V1.45 (Abramoff et al. [2004]).

All methods for growing plants followed NICS standards (NICS [2004]) and trait values were as described in Oh et al. ([2004]), Lee et al. ([2005]) and Jeong et al. ([2000]).

### Statistics of agronomic traits

Descriptive statistics including mean, median, and standard deviation were calculated. A paired t-test was used to compare the performance of 57 rice varieties under control and cold treated conditions in Chuncheon based on mean trait values for *DTH*, *CL*, *PN*, *SPP* and *PSS* for each variety. The Pearson correlation coefficient was used to estimate linear associations among phenotypic variables. Using ANOVA, the mean agronomic performance estimated for the group of 13 landraces and 67 improved varieties were also compared for each of the agronomic traits.

All statistical analyses were implemented by JMP Pro V9.0.2 (SAS Institute Inc.).

## Abbreviations

cpSSR: Chloroplast SSR: 

nSSR: Nuclear SSR: 

CL: Culm length: 

D_DTH: Difference in days to heading: 

D_PN: Difference in panicle number: 

DTH: Days to heading: 

GL: Grain length: 

GLWR: Grain length/width ratio: 

GT: Grain thickness: 

GW: Grain width: 

LDS: Leaf discoloration: 

PAM: Phenotype acceptability at maturity: 

PAT: Phenotype acceptability at tillering: 

PE: Panicle neck exertion: 

PN: Panicle number: 

PSS: Percentage of seed set: 

R_CL: Reduction ratio of culm length: 

R_PSS: Reduction ratio of percentage of seed set: 

R_SPP: Reduction ratio of spikelet number per panicle: 

SPP: Spikelet number per panicle: 

## Competing interests

The authors declare that they have no competing interest.

## Authors’ contributions

HK designed the project, performed the genotyping, evaluated grain traits, analyzed data and wrote the manuscript. EGJ and YJW generated the phenotypic data for the agronomic field trials and provided the DPRK rice germplasm to the project. SNA provided rice germplasm and critical insight during manuscript preparation. JD provided critical guidance for data analysis and manuscript preparation. NS participated in SSR genotyping. AJG assisted with analysis of phenotypic data. SRM provided intellectual vision and overall guidance for this project and helped with the organization and editing of the manuscript. All authors read and approved the final manuscript.

## Additional files

## Supplementary Material

Additional file 2: **Figure S1.** The value of delta K in structure analysis from K = 1 to K = 10 based on **(A)** DPRK germplasm, **(B)** Mini-Rice Diversity Panel and **(C)** combined DPRK germplasm and Mini-Rice Diversity Panel. **Figure S2**. **(A)** Unrooted genetic tree based on neighbor joining and **(B)** Principal component analysis based on DPRK rice germplasm and Mini-Rice Diversity Panel. Mini-Rice Diversity Panels are shown in circles; blue = *temperate japonica*; aqua = *tropical japonica*; pink = *aromatic*; red = *indica*; yellow = *aus*. DPRK accessions are presented as a branch without circle in **(A)** and in gray in **(B)**. **Figure S3**. Chloroplast haplotype network using sequence information and PCR amplicon length based on cpSSRs. Bars indicate mutation events and bar color indicates source of polymorphisms. **Figure S4**. Grain shape variation in DPRK rice germplasm. **Figure S5**. Phenotypic differences of improved varieties of *Group 1* between control and cold-water treated plot (17°C). Click here for file

Additional file 1: **Table S1.** Germplasm information and their genetic information based on Structure group and chloroplast haplotype. **Table S2**. Pairwise Fst among geographical group. **Table S3**. Information of molecular markers and polymorphic summary among DPRK accessions. **Table S4**. Pearson correlation coefficients of DPRK phenotypic performance. Click here for file
