# Supplementary material for: Nuclear and chloroplast diversity and phenotypic distribution of rice (Oryza sativa L.) germplasm from the democratic people’s republic of Korea (DPRK; North Korea)
Source: Rice (N Y). 2014 Jul 2;7(1):7. doi: 10.1186/s12284-014-0007-4 (PMC4078393; doi:10.1186/s12284-014-0007-4)

**Additional Files 1:**

Figure S1. The value of Delta K in structure analysis from K=1 to K=10 based on (A) DPRK germplasm, (B) Mini-Rice Diversity Panel and (C) combined DPRK germplasm and Mini-Rice Diversity Panel.

Figure S2. (A) Unrooted genetic tree based on neighbor joining and (B) Principal component analysis based on DPRK rice germplasm and Mini-Rice Diversity Panel. Mini-Rice Diversity Panels are shown in circles; blue = *temperate japonica*; aqua = *tropical japonica*; pink = *aromatic*; red = *indica*; yellow = *aus*. DPRK accessions are presented with without circle in (A) and in gray in (B).

Figure S3. Chloroplast haplotype network using sequence information and PCR amplicon length based on cpSSRs. Bars indicate mutation events and bar color indicates source of polymorphisms.

Figure S4. Grain shape variation in DPRK rice germplasm.

Figure S5. Phenotypic differences of *Group 1* varieties and *Group 2* varieties of DPRK between in control and cold-water treated plot (17°C).

Figure S1. The value of Delta K in structure analysis from K=1 to K=10 based on (A) DPRK germplasm, (B) Mini-Rice Diversity Panel and (C) combined DPRK germplasm and Mini-Rice Diversity Panel.

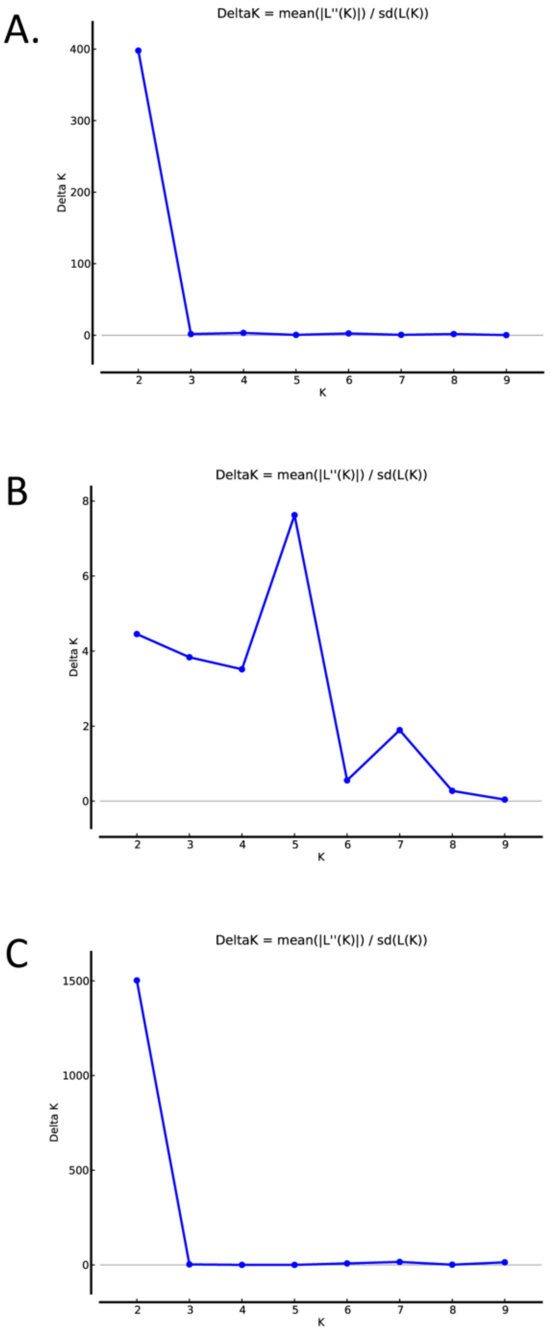

Figure S2. (A) Unrooted genetic tree based on neighbor joining and (B) Principal component analysis based on DPRK rice germplasm and Mini-Rice Diversity Panel. Mini-Rice Diversity Panels are shown in circles; blue = *temperate japonica*; aqua = *tropical japonica*; pink = *aromatic*; red = *indica*; yellow = *aus*. DPRK accessions are presented with without circle in (A) and in gray in (B).

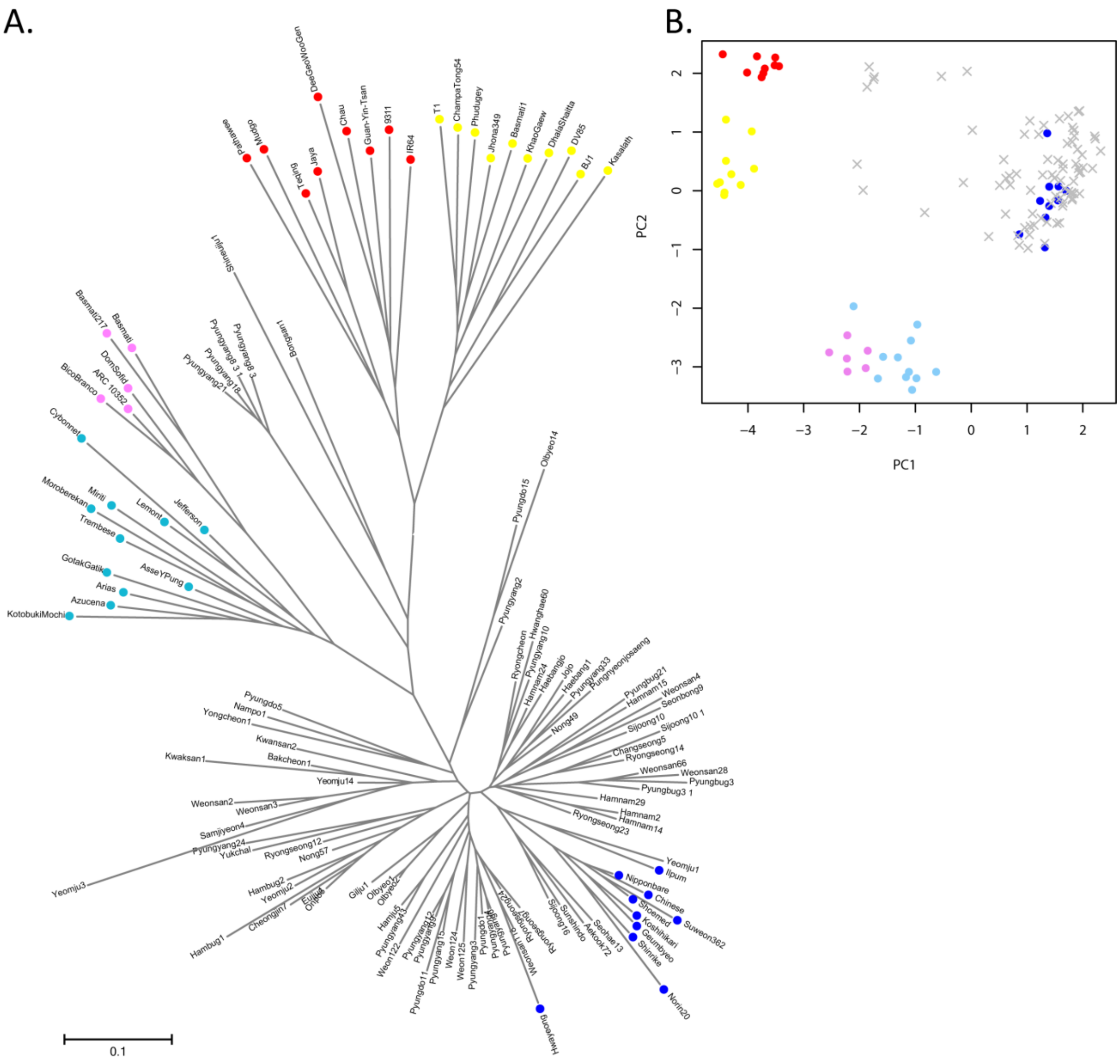

Figure S3. Chloroplast haplotype network using sequence information and PCR amplicon length based on cpSSRs. Bars indicate mutation events and bar color indicates source of polymorphisms.

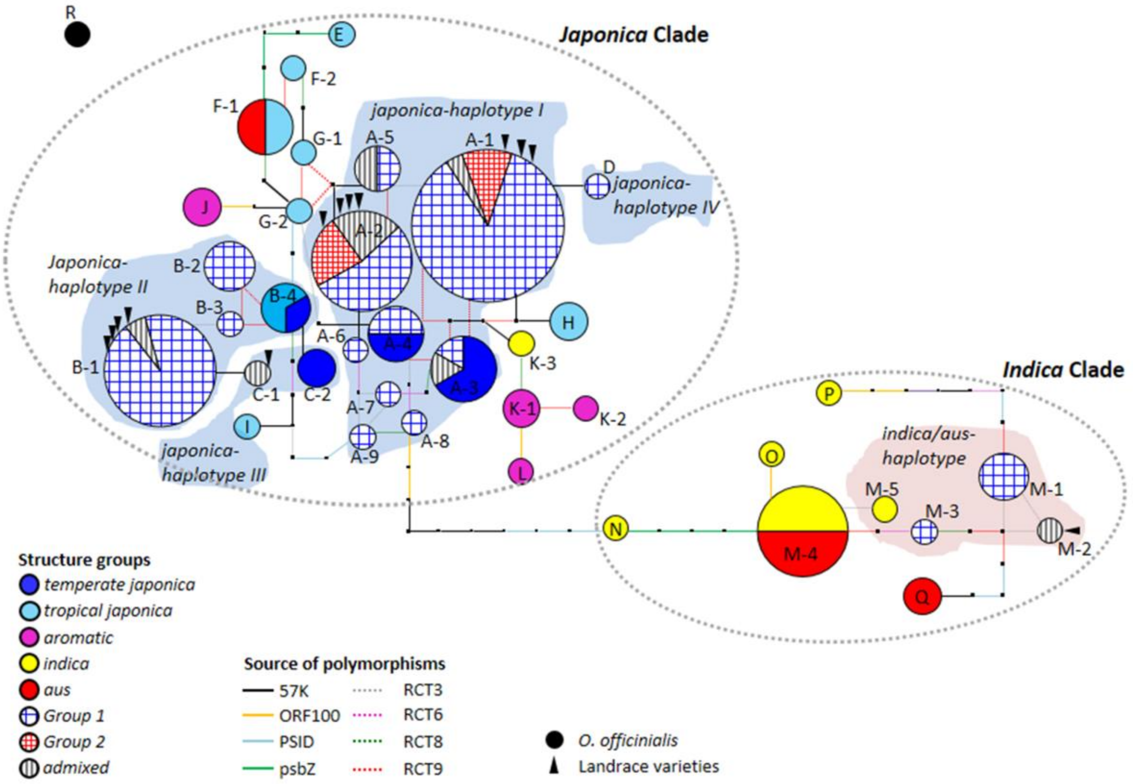

Figure S4. Grain shape variation in DPRK rice germplasm.

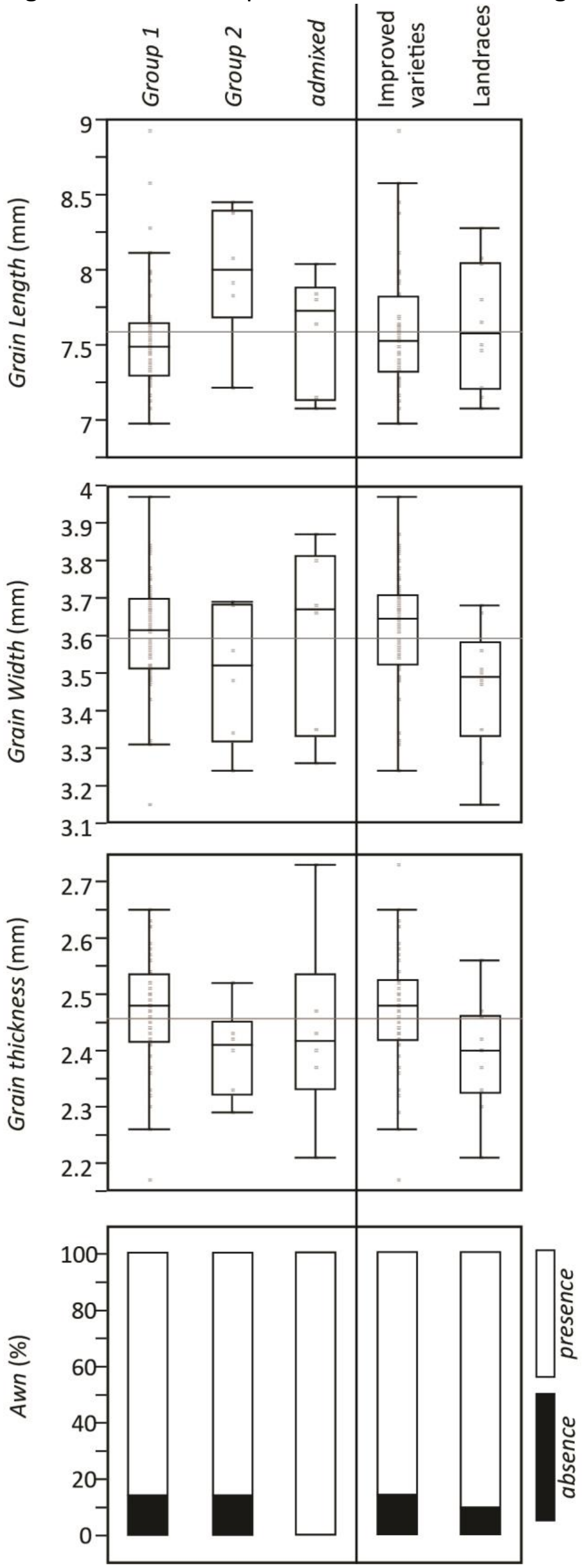

Figure S5. Phenotypic differences of improved varieties of *Group 1* between control and cold-water treated plot (17°C)

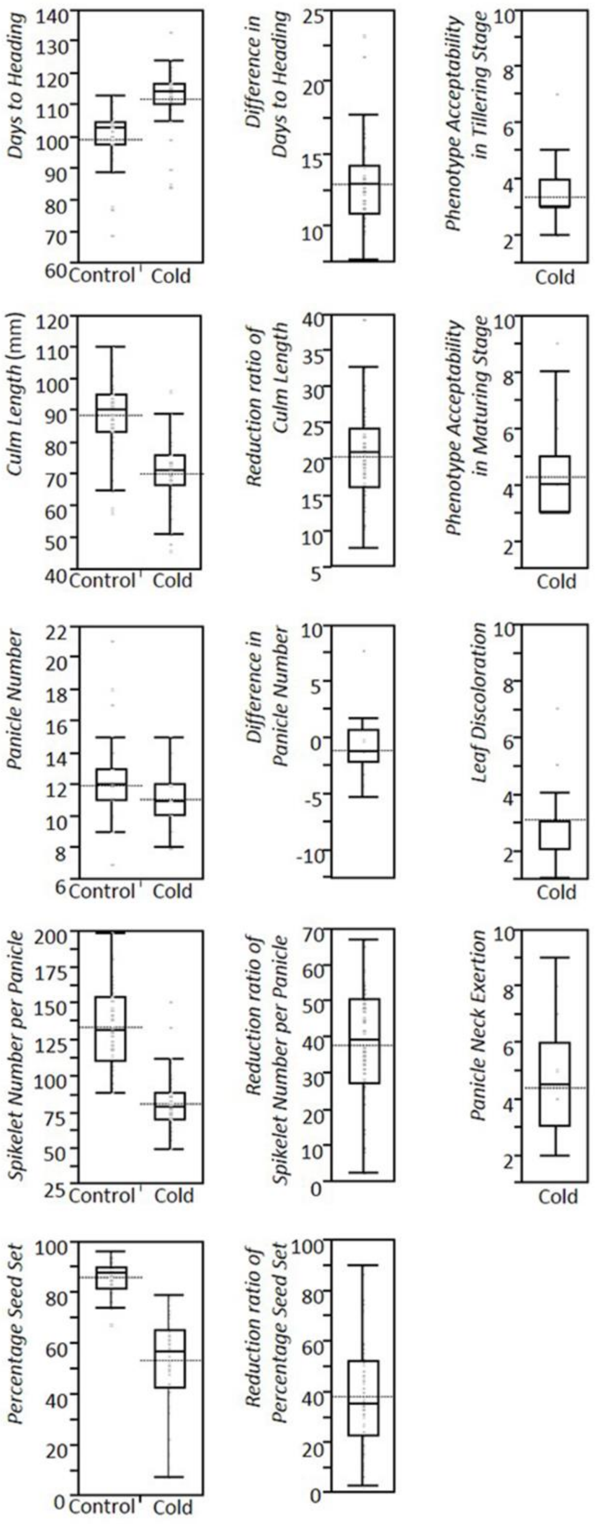

Supplement: Additional file 2: — Figure S1. The value of delta K in structure analysis from K = 1 to K = 10 based on (A) DPRK germplasm, (B) Mini-Rice Diversity Panel and (C) combined DPRK germplasm and Mini-Rice Diversity Panel. Figure S2. (A) Unrooted genetic tree based on neighbor joining and (B) Principal component analysis based on DPRK rice germplasm and Mini-Rice Diversity Panel. Mini-Rice Diversity Panels are shown in circles; blue = temperate japonica; aqua = tropical japonica; pink = aromatic; red = indica; yellow = aus. DPRK accessions are presented as a branch without circle in (A) and in gray in (B). Figure S3. Chloroplast haplotype network using sequence information and PCR amplicon length based on cpSSRs. Bars indicate mutation events and bar color indicates source of polymorphisms. Figure S4. Grain shape variation in DPRK rice germplasm. Figure S5. Phenotypic differences of improved varieties of Group 1 between control and cold-water treated plot (17°C). [file s12284-014-0007-4-S2.pdf]
