# Supplementary material for: Nuclear and chloroplast diversity and phenotypic distribution of rice (Oryza sativa L.) germplasm from the democratic people’s republic of Korea (DPRK; North Korea)
Source: Rice (N Y). 2014 Jul 2;7(1):7. doi: 10.1186/s12284-014-0007-4 (PMC4078393; doi:10.1186/s12284-014-0007-4)
Supplement: Additional file 1: — Table S1. Germplasm information and their genetic information based on Structure group and chloroplast haplotype. Table S2. Pairwise Fst among geographical group. Table S3. Information of molecular markers and polymorphic summary among DPRK accessions. Table S4. Pearson correlation coefficients of DPRK phenotypic performance. [file s12284-014-0007-4-S1.pdf]

Additional Files 2:

Table S1. Germplasm information and their genetic information based on Structure group and chloroplast haplotype

Table S2. Pairwise Fst among geographical group

Table S3. Information of molecular markers and polymorphic summary among DPRK accessions

Table S4. Pearson correlation coefficients of DPRK phenotypic performance

Table S1. Germplasm information and their genetic information based on Structure group and chloroplast haplotyp

I. DPRK germplasm

No.	Accession Name	Remark	Province in DPRK	Village/City	latitude	longitude	Percent ancestry *temperate japonica*	*indica*	Model based group in Figure 2A.	Chloroplast haplotype in Fig. 3	Chloroplast haplotype in Fig. S3
1	Nampo 1	landrace	Pyungannam-do	Nampo	38.73708	125.4	72.16	27.84	*admixed*	*M: indica/aus-haplotype*	*M-2*
2	Weonsan 2	landrace	Kangwon-do	Wonsan	39.1475	127.446111	69.92	30.08	*admixed*	*A: japonica-haplotype I*	*A-2*
3	Weonsan 3	landrace	Kangwon-do	Wonsan	39.1475	127.446111	98.61	1.39	*Group 1*	*A: japonica-haplotype I*	*A-1*
4	Weonsan 4	landrace	Kangwon-do	Wonsan	39.1475	127.446111	72.01	27.99	*admixed*	*A: japonica-haplotype I*	*A-2*
5	Shineuiju 1	landrace	Pyunganbuk-do	Shinuiju	40.18307	124.402397	0.20	99.80	*Group 2*	*A: japonica-haplotype I*	*A-1*
6	Yongcheon 1	landrace	Pyunganbuk-do	Ryongcheon	39.983333	124.466667	75.03	24.97	*admixed*	*C: japonica-haplotype III*	*C-1*
7	Euiju 1	landrace	Pyunganbuk-do	Uiju	40.196944	124.531944	99.80	0.20	*Group 1*	*B: japonica-haplotype II*	*B-1*
8	Cheongjin 7	landrace	Hamgyeongbuk-do	Cheongjin	41.78376	129.7836	97.09	2.91	*Group 1*	*B: japonica-haplotype II*	*B-1*
9	Hambuk 1	landrace	Hamgyeongbuk-do	-			48.38	51.62	*admixed*	*B: japonica-haplotype II*	*B-1*
10	Hambuk 2	landrace	Hamgyeongbuk-do	-			99.80	0.20	*Group 1*	*B: japonica-haplotype II*	*B-1*
11	Kwaksan 1	landrace	Pyunganbuk-do	Gwaksan	39.685129	125.086866	67.28	32.72	*admixed*	*A: japonica-haplotype I*	*A-2*
12	Bakcheon 1	landrace	Pyunganbuk-do	Bakcheon	39.635307	125.502319	95.53	4.47	*Group 1*	*A: japonica-haplotype I*	*A-1*
13	Bongsan 1	landrace	Hwanghaebuk-do	Bongsan	38.467763	125.861668	10.61	89.39	*Group 2*	*A: japonica-haplotype I*	*A-2*
14	Kwansan 2	improved varieties	Hwanghaenam-do	Kwansan	38.539573	125.249634	97.71	2.29	*Group 1*	*A: japonica-haplotype I*	*A-1*
15	Gilju 1	improved varieties	Hamgyeongbuk-do	Gilju	40.961944	129.336667	99.80	0.20	*Group 1*	*A: japonica-haplotype I*	*A-2*
16	Nong 49	improved varieties	-	-			99.80	0.20	*Group 1*	*A: japonica-haplotype I*	*A-1*
17	Nong 57	improved varieties	-	-			99.80	0.20	*Group 1*	*D: japonica-haplotype IV*	*D*
18	Ryongseong 7	improved varieties	Pyungannam-do	Ryongseong	39.044786	125.753765	99.83	0.17	*Group 1*	*A: japonica-haplotype I*	*A-2*
19	Ryongseong 12	improved varieties	Pyungannam-do	Ryongseong	39.044786	125.753765	99.80	0.20	*Group 1*	*A: japonica-haplotype I*	*A-1*
20	Ryongseong 14	improved varieties	Pyungannam-do	Ryongseong	39.044786	125.753765	99.90	0.10	*Group 1*	*A: japonica-haplotype I*	*A-1*
21	Ryongseong 23	improved varieties	Pyungannam-do	Ryongseong	39.044786	125.753765	99.70	0.30	*Group 1*	*A: japonica-haplotype I*	*A-1*
22	Ryongseong 24	improved varieties	Pyungannam-do	Ryongseong	39.044786	125.753765	99.90	0.10	*Group 1*	*B: japonica-haplotype II*	*B-1*
23	Ryongcheon	improved varieties	Pyunganbuk-do	Ryongcheon	39.983333	124.466667	99.80	0.20	*Group 1*	*A: japonica-haplotype I*	*A-1*
24	Samjiyeon 4	improved varieties	Yanggang-do	Samjiyeon	41.8	128.316667	90.50	9.50	*Group 1*	*B: japonica-haplotype II*	*B-1*
25	Seohae 13	improved varieties	-	-			99.83	0.17	*Group 1*	*B: japonica-haplotype II*	*B-1*
26	Seonbong 9	improved varieties	Hamgyeongbuk-do	Seonbong	42.354136	130.374883	98.88	1.12	*Group 1*	*A: japonica-haplotype I*	*A-1*
27	Sunshindo	improved varieties	-	-			99.80	0.20	*Group 1*	*A: japonica-haplotype I*	*A-2*
28	Sijung 10	improved varieties	Jagang-do	Sijung	41.054502	126.474609	83.29	16.71	*Group 1*	*A: japonica-haplotype I*	*A-1*
29	Sijung 10_1	improved varieties	Jagang-do	Sijung	41.054502	126.474609	99.80	0.20	*Group 1*	*A: japonica-haplotype I*	*A-1*
30	Sijung 16	improved varieties	Jagang-do	Sijung	41.054502	126.474609	99.80	0.20	*Group 1*	*A: japonica-haplotype I*	*A-3*
31	Aeguk 72	improved varieties	-	-			99.80	0.20	*Group 1*	*A: japonica-haplotype I*	*A-5*
32	Yeomju 1	improved varieties	Pyunganbuk-do	Yeomju	39.89056	124.603104	99.63	0.37	*Group 1*	*B: japonica-haplotype II*	*B-1*
33	Yeomju 2	improved varieties	Pyunganbuk-do	Yeomju	39.89056	124.603104	99.80	0.20	*Group 1*	*B: japonica-haplotype II*	*B-2*
34	Yeomju 3	improved varieties	Pyunganbuk-do	Yeomju	39.89056	124.603104	17.89	82.11	*Group 2*	-	-
35	Yeomju 14	improved varieties	Pyunganbuk-do	Yeomju	39.89056	124.603104	93.68	6.32	*Group 1*	*A: japonica-haplotype I*	*A-4*
36	Onpo 6	improved varieties	Hamgyeongbuk-do	Onpo	41.582958	129.602707	99.80	0.20	*Group 1*	*B: japonica-haplotype II*	*B-1*
37	Olbyeo 1	improved varieties	-	-			99.80	0.20	*Group 1*	*B: japonica-haplotype II*	*B-1*
38	Olbyeo 2	improved varieties	-	-			99.80	0.20	*Group 1*	*B: japonica-haplotype II*	*B-1*
39	Weon 122	improved varieties	-	-			99.80	0.20	*Group 1*	*A: japonica-haplotype I*	*A-2*
40	Weon 124	improved varieties	-	-			99.60	0.40	*Group 1*	*A: japonica-haplotype I*	*A-1*

41	Weon 125	improved varieties	-	-			99.80	0.20	Group 1	A: japonica-haplotype I	A-6
42	Weonsan 28	improved varieties	Kangwond-do	Wonsan	39.1475	127.446111	97.69	2.31	Group 1	A: japonica-haplotype I	A-8
43	Weonsan 66	improved varieties	Kangwond-do	Wonsan	39.1475	127.446111	99.80	0.20	Group 1	A: japonica-haplotype I	A-2
44	Weonsan 118	improved varieties	Kangwond-do	Wonsan	39.1475	127.446111	99.73	0.27	Group 1	A: japonica-haplotype I	A-1
45	Yukchal	improved varieties	-	-			80.64	19.36	Group 1	M: indica/aus-haplotype	M-1
46	Jojo	improved varieties	-	-			99.80	0.20	Group 1	-	-
47	Changseong 5	improved varieties	Pyunganbuk-do	Changseong	40.455468	125.213793	99.80	0.20	Group 1	A: japonica-haplotype I	A-1
48	Pyeongdo 1	improved varieties	-	-			99.60	0.40	Group 1	B: japonica-haplotype II	B-2
49	Pyeongbuk 3	improved varieties	Pyunganbuk-do	-			87.33	12.67	Group 1	A: japonica-haplotype I	A-1
50	Pyeongbuk 3_1	improved varieties	Pyunganbuk-do	-			99.75	0.25	Group 1	A: japonica-haplotype I	A-1
51	Pyeongbuk 21	improved varieties	Pyunganbuk-do	-			99.63	0.37	Group 1	A: japonica-haplotype I	A-1
52	Pyeongyang 2	improved varieties	Pyungannam-do	Pyeongyang	39.044786	125.753765	70.31	29.69	admixed	A: japonica-haplotype I	A-1
53	Pyeongyang 3	improved varieties	Pyungannam-do	Pyeongyang	39.044786	125.753765	99.73	0.27	Group 1	B: japonica-haplotype II	B-1
54	Pyeongyang 4	improved varieties	Pyungannam-do	Pyeongyang	39.044786	125.753765	99.85	0.15	Group 1	B: japonica-haplotype II	B-1
55	Pyeongyang 6	improved varieties	Pyungannam-do	Pyeongyang	39.044786	125.753765	99.85	0.15	Group 1	B: japonica-haplotype II	B-1
56	Pyeongyang 8_3	improved varieties	Pyungannam-do	Pyeongyang	39.044786	125.753765	0.20	99.80	Group 2	A: japonica-haplotype I	A-2
57	Pyeongyang 8_3_1	improved varieties	Pyungannam-do	Pyeongyang	39.044786	125.753765	0.20	99.80	Group 2	A: japonica-haplotype I	A-2
58	Pyeongyang 9	improved varieties	Pyungannam-do	Pyeongyang	39.044786	125.753765	99.80	0.20	Group 1	A: japonica-haplotype I	A-2
59	Pyeongyang 10	improved varieties	Pyungannam-do	Pyeongyang	39.044786	125.753765	99.80	0.20	Group 1	A: japonica-haplotype I	A-2
60	Pyeongyang 12	improved varieties	Pyungannam-do	Pyeongyang	39.044786	125.753765	99.83	0.17	Group 1	B: japonica-haplotype II	B-1
61	Pyeongyang 15	improved varieties	Pyungannam-do	Pyeongyang	39.044786	125.753765	99.80	0.20	Group 1	B: japonica-haplotype II	B-2
62	Pyeongyang 18	improved varieties	Pyungannam-do	Pyeongyang	39.044786	125.753765	0.20	99.80	Group 2	A: japonica-haplotype I	A-1
63	Pyeongyang 21	improved varieties	Pyungannam-do	Pyeongyang	39.044786	125.753765	0.20	99.80	Group 2	A: japonica-haplotype I	A-1
64	Pyeongyang 24	improved varieties	Pyungannam-do	Pyeongyang	39.044786	125.753765	91.87	8.13	Group 1	M: indica/aus-haplotype	M-3
65	Pyeongyang 33	improved varieties	Pyungannam-do	Pyeongyang	39.044786	125.753765	99.80	0.20	Group 1	A: japonica-haplotype I	A-1
66	Pyeongyangjosaeng	improved varieties	Pyungannam-do	Pyeongyang	39.044786	125.753765	89.25	10.75	Group 1	A: japonica-haplotype I	A-1
67	Hamnam 2	improved varieties	Hamgyeongnam-do	-			99.44	0.56	Group 1	A: japonica-haplotype I	A-1
68	Hamnam 14	improved varieties	Hamgyeongnam-do	-			99.80	0.20	Group 1	A: japonica-haplotype I	A-1
69	Hamnam 15	improved varieties	Hamgyeongnam-do	-			99.80	0.20	Group 1	M: indica/aus-haplotype	M-1
70	Hamnam 24	improved varieties	Hamgyeongnam-do	-			99.60	0.40	Group 1	A: japonica-haplotype I	A-1
71	Hamnam 29	improved varieties	Hamgyeongnam-do	-			99.80	0.20	Group 1	A: japonica-haplotype I	A-1
72	Hamju 5	improved varieties	Hamgyeongnam-do	Hamju	39.846775	127.437013	99.33	0.67	Group 1	B: japonica-haplotype II	B-3
73	Haebang 1	improved varieties	-	-			99.80	0.20	Group 1	A: japonica-haplotype I	A-7
74	Haebangjo	improved varieties	-	-			99.81	0.19	Group 1	A: japonica-haplotype I	A-9
75	Hwanghae 60	improved varieties	Hwanghae-do	-			99.80	0.20	Group 1	A: japonica-haplotype I	A-4
76	Pyeongyang 43	improved varieties	Pyungannam-do	Pyeongyang	39.044786	125.753765	99.24	0.76	Group 1	B: japonica-haplotype II	B-1
77	Pyeongdo 5	improved varieties	-	-			69.38	30.62	admixed	M: indica/aus-haplotype	M-1
78	Pyeongdo 11	improved varieties	-	-			99.70	0.30	Group 1	A: japonica-haplotype I	A-1
79	Pyeongdo 15	improved varieties	-	-			45.43	54.57	admixed	A: japonica-haplotype I	A-5
80	Olbyeo 14	improved varieties	-	-			29.76	70.24	admixed	A: japonica-haplotype I	A-3

II. Mini-Rice Diversity Pane

No.	Accession Name	Country of Origin	Percent ancestry					Model based group in Figure 2B.	Chloroplast haplotype in Fig. 3	Chloroplast haplotype in Fig. S3
			temperate japonica	tropical japonica	aromatic	indica	aus			
1	Chinese	China	99.89	0.10	0.01	0.00	0.00	temperate japonica	A: japonica-haplotype I	A-3

2	Shoemed	USA	99.89	0.10	0.01	0.00	0.00	*temperate japonica*	*B: japonica-haplotype II*	*B-4*
3	Koshihikari	Japan	99.80	0.10	0.03	0.04	0.03	*temperate japonica*	*A: japonica-haplotype I*	*A-4*
4	Suweon 362	South Korea	99.78	0.10	0.02	0.00	0.10	*temperate japonica*	*C: japonica-haplotype III*	*C-2*
5	Nipponbare	Japan	99.51	0.10	0.39	0.00	0.00	*temperate japonica*	*A: japonica-haplotype I*	*A-3*
6	Geumbyeo	South Korea	99.43	0.37	0.09	0.01	0.10	*temperate japonica*	*A: japonica-haplotype I*	*A-4*
7	Shinrike	Japan	98.94	0.95	0.11	0.00	0.00	*temperate japonica*	*A: japonica-haplotype I*	*A-3*
8	Hwayeong	South Korea	92.05	0.04	7.72	0.10	0.09	*temperate japonica*	*C: japonica-haplotype III*	*C-2*
9	Ilpum	South Korea	91.31	0.10	8.41	0.09	0.09	*temperate japonica*	-	-
10	Norin 20	Japan	84.50	15.04	0.43	0.03	0.00	*temperate japonica*	*A: japonica-haplotype I*	*A-3*
11	Azucena	Philippines	0.14	99.76	0.09	0.01	0.00	*tropical japonica*	*I*	*I*
12	Lemont	USA	0.12	99.71	0.15	0.02	0.00	*tropical japonica*	*F*	*F-1*
13	Moroberekan	Guinea	0.11	99.57	0.10	0.00	0.22	*tropical japonica*	*B: japonica-haplotype II*	*B-4*
14	Miriti	Bangladesh	0.10	99.26	0.35	0.19	0.10	*tropical japonica*	*H*	*H*
15	Trembese	Indonesia	0.48	99.17	0.33	0.01	0.01	*tropical japonica*	*G*	*G-2*
16	Kotobuki Mochi	Japan	0.10	99.13	0.19	0.10	0.48	*tropical japonica*	*F*	*F-2*
17	Arias	Indonesia	0.70	98.92	0.27	0.01	0.10	*tropical japonica*	*H*	*H*
18	Gotak Gatik	Indonesia	0.40	98.89	0.53	0.08	0.10	*tropical japonica*	*G*	*G-1*
19	Cybonnet	USA	0.34	97.65	0.70	1.18	0.13	*tropical japonica*	*E*	*E*
20	Asse Y Pung	Philippines	7.17	92.60	0.12	0.01	0.10	*tropical japonica*	*B: japonica-haplotype II*	*B-4*
21	Jefferson	USA	12.07	87.42	0.22	0.18	0.11	*tropical japonica*	*F*	*F-1*
22	Basmati	Pakistan	0.02	16.11	79.86	0.01	4.00	*aromatic*	*K*	*K-1*
23	ARC_10352	India	0.10	20.02	79.84	0.00	0.04	*aromatic*	*L*	*L*
24	Dom Sofid	Iran	0.09	20.01	79.78	0.02	0.10	*aromatic*	*J*	*J*
25	Bico Branco	Brazil	0.10	19.90	79.71	0.10	0.19	*aromatic*	*J*	*J*
26	Basmati 217	India	0.24	13.98	79.02	0.10	6.66	*aromatic*	*K*	*K-1*
27	Kitrana 508	Madagascar	0.10	54.69	44.99	0.10	0.12	*admixed*	*K*	*K-2*
28	Pathawee	Sri Lanka	0.00	0.10	0.12	99.68	0.10	*indica*	*F*	*F-1*
29	Teqing	China	0.10	0.10	0.06	99.64	0.10	*indica*	*Q*	*Q*
30	Jaya	India	0.10	0.10	0.19	99.51	0.10	*indica*	*M: indica/aus-haplotype*	*M-4*
31	Mudgo	India	0.10	0.10	0.59	97.90	1.31	*indica*	*M: indica/aus-haplotype*	*M-4*
32	Dee Geo Woo Gen	Taiwan	0.00	0.00	7.85	91.73	0.42	*indica*	*M: indica/aus-haplotype*	*M-4*
33	Chau	Vietnam	0.02	0.10	8.29	91.48	0.11	*indica*	*Q*	*Q*
34	9311	China	0.10	0.00	8.65	91.15	0.10	*indica*	*M: indica/aus-haplotype*	*M-4*
35	Guan-Yin-Tsan	China	0.10	0.10	8.73	90.97	0.10	*indica*	*M: indica/aus-haplotype*	*M-4*
36	IR64	Philippines	0.25	0.10	8.73	90.82	0.10	*indica*	*F*	*F-1*
37	Khao Gaew	Thailand	0.00	0.10	0.03	0.10	99.77	*aus*	*M: indica/aus-haplotype*	*M-4*
38	Champa Tong 54	Thailand	0.00	0.14	0.10	0.10	99.66	*aus*	*O*	*O*
39	Basmati1	Pakistan	0.00	0.04	0.21	0.10	99.65	*aus*	*M: indica/aus-haplotype*	*M-4*
40	Phudugey	Bhutan	0.10	0.10	0.10	0.06	99.64	*aus*	*M: indica/aus-haplotype*	*M-4*
41	Jhona 349	India	0.00	0.07	0.22	0.10	99.61	*aus*	*M: indica/aus-haplotype*	*M-4*
42	T 1	India	0.10	0.10	0.11	0.10	99.59	*aus*	*N*	*N*
43	Dhala Shaitta	Bangladesh	0.10	0.10	0.50	0.20	99.10	*aus*	*M: indica/aus-haplotype*	*M-4*
44	Kasalath	India	0.10	0.00	1.09	0.19	98.62	*aus*	*M: indica/aus-haplotype*	*M-5*
45	DV85	Bangladesh	0.00	0.00	1.32	0.10	98.58	*aus*	*P*	*P*
46	BJ 1	India	0.10	0.10	1.48	0.19	98.13	*aus*	*K*	*K-3*

III. Out-group

No.	Accession Name	Remark	Country of Origin	Chloroplast haplotype in Fig. 3	Chloroplast haplotype in Fig. S3
1	*O.officinialis*	IRGC 105220	Indonesia	*R*	*R*

Table S2. Pairwise Fst among geographical group

Province*	Hamgyeongbuk-do	Hamgyeongnam-do	Pyunganbuk-do	Pyungannam-do	Hwanghae-do	Kangwon-do	unknown
Hamgyeongbuk-do	0						
Hamgyeongnam-do	0.23396*	0					
Pyunganbuk-do	0.07942	0.0909	0				
Pyungannam-do	0.14462*	0.0919	0.07202	0			
Hwanghae-do	0.22121	0.23689**	0.12738	0.13538	0		
Kangwon-do	0.15808*	0.20872**	0.06929	0.1249*	0.20588	0	

* Province was shown in Figure 1.

Table S3. Information of molecular markers and polymorphic summary among DPRK accessions

I. Nuclear markers

No. o	Marker ID	Marker Type	Location	Location	Motif	Forward Primer	Reverse Primer	Frequency of Major Allele	No. of Allele	Gene Diversity	PIC	Remark
1	RM1	nSSR	Chr.1_4,636,870-4,636,793	Chr.1	(GA)26	GCGAAAACACAATGCAAAAA	GCGTTGGTTGGACCTGAC	0.6104	5	0.5205	0.4405	
2	RM283	nSSR	Chr.1_4,887,070-4,886,915	Chr.1	(GA)18	GTCTACATGTACCCTTGTTGGG	CGGCATGAGAGTCTGTGATG	0.4937	7	0.5896	0.5076	*
3	RM312	nSSR	Chr.1_10,985,604-10,985,504	Chr.1	(ATTT)4(GT)9	GTATGCATATTTGATAAGAG	AAGTCACCGAGTTTACCTTC	0.8101	3	0.3249	0.3002	*
4	RM5	nSSR	Chr.1_23,972,524-23,972,412	Chr.1	(GA)14	TGCAACTTCTAGCTGCTCGA	GCATCCGATCTTGATGGG	0.4375	7	0.6753	0.6216	
5	RM237	nSSR	Chr.1_30,027,538-30,027,408	Chr.1	(CT)18	CAAATCCCGACTGCTGTCC	TGGGAAGAGAGCACTACAGC	0.4875	38	0.7538	0.7496	
6	RM431	nSSR	Chr.1_42,553,316-42,553,064	Chr.1	(AG)16	TCCTGCGAACTGAAGAGTTG	AGAGCAAAACCCTGGTTCAC	0.8718	5	0.2314	0.2184	
7	RM154	nSSR	Chr.2_1,084,077-1,083,895	Chr.2	(GA)21	ACCCTCTCCGCCTCGCCTCCTC	CTCCTCCTCCTGCGACCGCTCC	0.7436	9	0.4352	0.4221	
8	RM279	nSSR	Chr.2_2,882,217-2,882,052	Chr.2	(GA)16	GCGGGAGAGGGATCTCCT	GGCTAGGAGTTAACCTCGCG	0.9500	4	0.0966	0.0949	
9	RM452	nSSR	Chr.2_9,563,514-9,563,308	Chr.2	(GTC)9	CTGATCGAGAGCGTTAAGGG	GGGATCAAACCACGTTTCTG	0.9867	2	0.0263	0.0260	
10	RM208	nSSR	Chr.2_37,297,032-37,296,854	Chr.2	(CT)17	TCTGCAAGCCTTGTCTGATG	TAAGTCGATCATTGTGTGGACC	0.4416	3	0.6187	0.5381	
11	RM22	nSSR	Chr.3_1,747,654-1,747,462	Chr.3	(GA)22	GGTTTGGGAGCCCATAATCT	CTGGGCTTCTTTCACTCGTC	0.6625	4	0.4947	0.4359	
12	RM489	nSSR	Chr.3_4,734,471-4,734,237	Chr.3	(ATA)8	ACTTGAGACGATCGGACACC	TCACCCATGGATGTTGTCAG	0.9114	6	0.1676	0.1647	*
13	OSR13	nSSR	Chr.3_7,609,918-7,609,824	Chr.3	(GA)n	CATTTGTGCGTCACGGAGTA	AGCCACAGCGCCCATCTCTC	0.3544	6	0.7226	0.6759	*
14	RM338	nSSR	Chr.3_14,850,095-14,849,912	Chr.3	(CTT)6	CACAGGAGCAGGAGAAGAGC	GGCAAACCGATCACTCAGTC	0.9750	2	0.0488	0.0476	
15	RM514	nSSR	Chr.3_39,644,541-39,644,293	Chr.3	(AC)12	AGATTGATCTCCCATTCCCC	CACGAGCATATTACTAGTGG	0.8831	2	0.2064	0.1851	*
16	RM261	nSSR	Chr.4_5,458,470-5,458,347	Chr.4	(C)9(CT)8	CTACTTCTCCCCTTGTGTCG	TGTACCATCGCCAAATCTCC	0.9875	2	0.0247	0.0244	
17	RM142	nSSR	Chr.4_18,571,830-18,571,592	Chr.4	(CGG)7	CTCGCTATCGCCATCGCCATCG	TCGAGCCATCGCTGGATGGAGG	0.8590	4	0.2535	0.2406	
18	RM124	nSSR	Chr.4_34,081,311-34,081,047	Chr.4	(TC)10	ATCGTCTGCGTTGCGGCTGCTG	CATGGATCACCGAGCTCCCCCC	0.9000	5	0.1856	0.1785	
19	RM507	nSSR	Chr.5_102,999-102,742	Chr.5	(AAGA)7	CTTAAGCTCCAGCCGAAATG	CTCACCCTCATCATCGCC	0.9200	2	0.1472	0.1364	
20	RM413	nSSR	Chr.5_2,212,839-2,212,736	Chr.5	(AG)11	GGCGATTCTTGGATGAAGAG	TCCCCACCAATCTTGTCTTC	0.9000	4	0.1859	0.1793	
21	RM169	nSSR	Chr.5_7,498,084-7,497,976	Chr.5	(GA)12	TGGCTGGCTCCGTGGGTAGCTG	TCCCGTTGCCGTTCATCCCTCC	0.6750	5	0.4738	0.4092	
22	RM161	nSSR	Chr.5_20,903,469-20,903,286	Chr.5	(AG)20	TGCAGATGAGAAGCGGCGCCTC	TGTGTCATCAGACGGCGCTCCG	0.4026	5	0.6601	0.5973	
23	RM178	nSSR	Chr.5_26,632,046-26,631,928	Chr.5	(GA)5(AG)8	TCGCGTGAAAGATAAGCGGCGC	GATCACCGTTCCCTCCGCCTGC	0.9481	2	0.0985	0.0936	
24	RM133	nSSR	Chr.6_260,690-260,468	Chr.6	(CT)8	TTGGATTGTTTTGCTGGCTCGC	GGAACACGGGGTCGGAAGCGAC	0.9054	4	0.1753	0.1672	
25	RM136	nSSR	Chr.6_9,683,725-9,683,621	Chr.6	(AGG)7	GAGAGCTCAGCTGCTGCCTCTAGC	GAGGAGCGCCACGGTGTACGCC	0.8987	3	0.1859	0.1755	
26	RM454	nSSR	Chr.6_24,277,299-24,277,034	Chr.6	(GCT)8	CTCAAGCTTAGCTGCTGCTG	GTGATCAGTGCACCATAGCG	0.5443	4	0.5701	0.4889	
27	RM162	nSSR	Chr.6_24,975,506-24,975,296	Chr.6	(AC)20	GCCAGCAAAACCAGGGATCCGG	CAAGGTCTTGTGCGGCTTGCGG	0.4750	9	0.7175	0.6896	
28	RM125	nSSR	Chr.7_5,359,588-5,359,464	Chr.7	(GCT)8	ATCAGCAGCCATGGCAGCGACC	AGGGGATCATGTGCCGAAGGCC	0.4625	5	0.6088	0.5304	
29	RM11	nSSR	Chr.7_17,422,404-17,422,259	Chr.7	(GA)17	TCTCCTCTTCCCCCGATC	ATAGCGGGCGAGGCTTAG	0.8481	5	0.2746	0.2659	
30	RM118	nSSR	Chr.7_24,655,977-24,655,819	Chr.7	(GA)8	CCAATCGGAGCCACCGGAGAGC	CACATCCTCCAGCGACGCCGAG	0.9250	4	0.1416	0.1367	
31	RM408	nSSR	Chr.8_126,496-126,372	Chr.8	(CT)13	CAACGAGCTAACTTCCGTCC	ACTGCTACTTGGGTAGCTGACC	0.7368	5	0.4269	0.3940	
32	RM152	nSSR	Chr.8_684,114-683,963	Chr.8	(GGC)10	GAAACCACCACACCTCACCG	CCGTAGACCTTCTTGAAGTAG	0.8630	4	0.2473	0.2356	
33	RM25	nSSR	Chr.8_4,644,995-4,644,693	Chr.8	(GA)18	GGAAAGAATGATCTTTTCATGG	CTACCATCAAAACCAATGTTC	0.8875	6	0.2088	0.2031	*
34	RM310	nSSR	Chr.8_5,381,388-5,381,304	Chr.8	(GT)19	CCAAAACATTTAAAATATCATG	GCTTGTTGGTCATTACCATTC	0.8250	5	0.3091	0.2950	
35	RM44	nSSR	Chr.8_11,759,506-11,759,388	Chr.8	(GA)16	ACGGGCAATCCGAACAACC	TCGGGAAAACCTACCCTACC	0.8101	9	0.3378	0.3300	
36	RM284	nSSR	Chr.8_22,479,322-22,479,177	Chr.8	(GA)8	ATCTCTGATACTCCATCCATCC	CCTGTACGTTGATCCGAAGC	0.3205	8	0.7811	0.7489	
37	RM433	nSSR	Chr.8_25,824,313-25,824,088	Chr.8	(AG)13	TGCGCTGAACTAAACACAGC	AGACAAACCTGGCCATTCAC	0.8289	3	0.2981	0.2778	*
38	RM447	nSSR	Chr.8_28,328,333-28,328,217	Chr.8	(CTT)8	CCCTTGTGCTGTCTCCTCTC	ACGGGCTTCTTCTCCTTCTC	0.8205	6	0.3156	0.3005	
39	RM316	nSSR	Chr.9_781,826-781,627	Chr.9	(GT)8-(TG)9	CTAGTTGGGCATACGATGGC	ACGCTTATATGTTACGTCAAC	0.7534	5	0.3944	0.3513	
40	RM105	nSSR	Chr.9_11,018,051-11,017,920	Chr.9	(CCT)6	GTCGTCGACCCATCGGAGCCAC	TGGTCGAGGTGGGGATCGGGTC	0.8125	5	0.3200	0.2938	*
41	RM215	nSSR	Chr.9_21,189,603-21,189,232	Chr.9	(CT)16	CAAAATGGAGCAGCAAGAGC	TGAGCACCTCCTTCTCTGTAG	0.4933	4	0.6393	0.5755	
42	RM474	nSSR	Chr.10_1,763,614-1,763,390	Chr.10	(AT)13	AAGATGTACGGGTGGCATTC	TATGAGCTGGTGAGCAATGG	0.2308	11	0.8287	0.8068	
43	RM271	nSSR	Chr.10_3,133,393-3,133,307	Chr.10	(GA)15	TCAGATCTACAATTCCATCC	TCGGTGAGACCTAGAGAGCC	0.6301	3	0.5337	0.4764	
44	RM484	nSSR	Chr.10_21138548-21138250	Chr.10	(AT)9	TCTCCCTCCTCACCATTGTC	TGCTGCCCTCTCTCTCTCTC	0.8312	3	0.2847	0.2509	
45	RM116	nSSR	Chr.11_5,745,452-5,745,174	Chr.11	(CT)9	TCACGCACAGCGTGCCGTTCTC	CAAGATCAAGCCATGAAAGGAGGG	0.8947	3	0.1908	0.1769	
46	RM536	nSSR	Chr.11_8990776-8990534	Chr.11	(CT)16	TCTCTCCTCTTGTTTGGCTC	ACACACCAACACGACCACAC	0.6329	5	0.5307	0.4750	
47	RM287	nSSR	Chr.11_17,233,680-17,233,574	Chr.11	(GA)21	TTCCCTGTTAAGAGAGAAATC	GTGTATTTGGTGAAAGCAAC	0.9091	4	0.1700	0.1642	
48	RM144	nSSR	Chr.11_28,805,157-28,804,933	Chr.11	(ATT)11	TGCCCTGGCGCAAATTTGATCC	GCTAGAGGAGATCAGATGGTAGTGCATG	0.3000	11	0.7603	0.7217	*
49	RM19	nSSR	Chr.12_2,607,554-2,607,276	Chr.12	(ATC)10	CAAAAACAGAGCAGATGAC	CTCAAGATGGACGCCAAGA	0.8800	4	0.2165	0.2025	*

50	RM512	nSSR	Chr.12_5,105,497-5,105,284	Chr.12	(TTTA)5	CTGCCTTTCTTACCCCCTTC	AACCCCTCGCTGGATTCTAG	0.8684	3	0.2316	0.2102	*
51	RM277	nSSR	Chr.12_18,319,138-18,319,022	Chr.12	(GA)11	CGGTCAAATCATCACCTGAC	CAAGGCTTGCAAGGGAAG	0.8354	3	0.2788	0.2464	

II. Chloroplast markers

No.	Marker ID	Marker Type	Location (bp on NC 001320)	Motif	Forward Primer	Reverse Primer	Frequency of Major Allele	No. of Allele	GeneDiversity	PIC	Remark
	ORF100		7888-8804		GCCGCTTTAGTCCACTCAGCCATC	TCAATGCCTTTTTTCAATGGTCTC					
52	ORF100_1	cpSSR	8003-8009	(A)7			1.0000	1.0000	0.0000	0.0000	
53	ORF100_2	SNP	8026				1.0000	1.0000	0.0000	0.0000	
54	ORF100_3	SNP	8127				0.9359	2.0000	0.1200	0.1128	
55	ORF100_5	SNP	8147				1.0000	1.0000	0.0000	0.0000	
56	ORF100_7	SNP	8193				1.0000	1.0000	0.0000	0.0000	
57	ORF100_8	Indel	8211-8212				1.0000	1.0000	0.0000	0.0000	
58	ORF100_9	SNP	8396				1.0000	1.0000	0.0000	0.0000	
59	ORF100_11	SNP	8415				1.0000	1.0000	0.0000	0.0000	
60	ORF100_12	SNP	8425				1.0000	1.0000	0.0000	0.0000	
61	ORF100_10	Indel	8430-8431				1.0000	1.0000	0.0000	0.0000	
62	ORF100_14	Indel	8537-8538				1.0000	1.0000	0.0000	0.0000	
63	ORF100_16	Indel	8538-8539				1.0000	1.0000	0.0000	0.0000	
64	ORF100_17	Indel	8548-8616				0.9359	2.0000	0.1200	0.1128	
65	ORF100_18_1	SNP	8599				0.9359	2.0000	0.1200	0.1128	
66	ORF100_19	SNP	8631				1.0000	1.0000	0.0000	0.0000	
67	ORF100_20	SNP	8720				1.0000	1.0000	0.0000	0.0000	
68	ORF100_21	SNP	8721				1.0000	1.0000	0.0000	0.0000	
	PSBZ		12013-12913		TATTTGCTTCTCCTGATGGTTGGT	GAGCGGAGTAGAGCAGTTTGGTAG					
69	psbZ_2	SNP	12210				1.0000	1.0000	0.0000	0.0000	
70	psbZ_4	Indel	12309-12310				1.0000	1.0000	0.0000	0.0000	
71	psbZ_7	Indel	12483				1.0000	1.0000	0.0000	0.0000	
72	psbZ_8	SNP	12496				0.9359	2.0000	0.1200	0.1128	
73	psbZ_9	Indel	12501-12502				1.0000	1.0000	0.0000	0.0000	
74	psbZ_11	SNP	12561				1.0000	1.0000	0.0000	0.0000	
75	psbZ_12	Indel	12672-12675				0.9359	2.0000	0.1200	0.1128	
76	psbZ_13	SNP	12677				1.0000	1.0000	0.0000	0.0000	
77	psbZ_14	SNP	12701				1.0000	1.0000	0.0000	0.0000	
78	psbZ_16	SNP	12741				1.0000	1.0000	0.0000	0.0000	
79	psbZ_17	SNP	12799				0.9359	2.0000	0.1200	0.1128	
80	psbZ_18	2bp substitution	12819-12820				0.9359	2.0000	0.1200	0.1128	
	57K		56210-57987		AGAATCTGGACCCATCGT	CGAATCGGTCATAACCAC					
81	57K_2	cpSSR	57009-57017	(T)9			1.0000	1.0000	0.0000	0.0000	
82	57K_3	Indel	57024-57076								
83	57K_4	Indel	57026-57041				0.9359	2.0000	0.1200	0.1128	
84	57K_5_1	2bp substitution	57042-57043				0.6667	2.0000	0.4444	0.3457	
85	57K_6	cpSSR	57052-57060	(A)8			0.9359	2.0000	0.1200	0.1128	
86	57K_8_1	SNP	57069				0.9744	2.0000	0.0500	0.0487	
87	57K_7	Indel	57070-57071				0.9359	2.0000	0.1200	0.1128	
88	57K_9	SNP	57114				1.0000	1.0000	0.0000	0.0000	
89	57K_10	Indel	57135-57136				1.0000	1.0000	0.0000	0.0000	
90	57K_12	SNP	57165				1.0000	1.0000	0.0000	0.0000	
91	57K_13	SNP	57174				1.0000	1.0000	0.0000	0.0000	
	PS-ID		77584-78118		ATCTGCAGCATTTAAAAGGG	AAAGATCTAGATTTCGTAAACA					
92	PS-ID_2	compound	77716-77722	(T)7	TCTGAGGTTGATCAT	ACATAGAGGAAGAA	0.6667	3.0000	0.4790	0.4103	
93	PS-ID_3	cpSSR	77723-77728	(G)6			0.6667	3.0000	0.4790	0.4103	
94	PS-ID_6	SNP	77793				0.9359	2.0000	0.1200	0.1128	

95	PS-ID_7	SNP	77903				1.0000	1.0000	0.0000	0.0000
96	PS-ID_8	SNP	77906				1.0000	1.0000	0.0000	0.0000
97	PS-ID_9	SNP	78064				1.0000	1.0000	0.0000	0.0000
98	RCT1	cpSSR	3496-3598	(A)10	CATCCTTTTCAATCCAAAATCA	TGCCTGATGTAGGGAAAAGC	1.0000	1.0000	0.0000	0.0000
99	RCT3	cpSSR	43831-43959	(A)10	TAGGCATAATTCCCAACCCA	CTTATCCATTTGGAGCATAGGG	0.6883	3.0000	0.4503	0.3782
100	RCT6	cpSSR	76190-76300	(A)10	GAATTTTAGAACTTTGAATTTTTTACCC	AAGCGTACCGAAGACTCGAA	0.9600	2.0000	0.0768	0.0739
101	RCT8	cpSSR	78336-78466	(T)17	ATAGTCAAGAAAGAGGATCTAGAAT	ACCGCGATTCAATAAGAGTA	0.9487	2.0000	0.0973	0.0926
102	RCT9	cpSSR	80571-80714	(T)10	ATAAGGTTATTCCCCGCTTACC	AAATTGGGGGAATTCGTACC	0.6933	3.0000	0.4523	0.3875
103	rps8(76K)	Indel	76702-76703		AAAACGTTGTATTTTTGTTT	TTCTCGAGGTATAATGACAG	1.0000	1.0000	0.0000	0.0000

* additional markers used only in 80 DPRK accessions

Table S4. Pearson correlation coefficients of DPRK phenotypic performance

		Control plot										Cold water irrigated plot									Phenotype Differences between control and cold water irrigated plots				
	Trait	*GL*	*GW*	*GT*	*GLWR*	*AWN*	*DTH*	*CL*	*PN*	*SPP*	*PSS*	*DTH*	*CL*	*PN*	*SPP*	*PSS*	*LDS*	*PE*	*PAT*	*PAM*	*D_DTH*	*R_CL*	*D_PN*	*R_SPP*	*R_PSS*
Control plot	*GL*	1																							
	GW	0.128	1																						
	GT	0.179	0.292	1																					
	GLWR	0.797**	-0.495**	-0.025	1																				
	AWN	-0.012	0.077	-0.003	-0.055	1																			
	DTH	-0.182	-0.104	0.145	-0.095	0.135	1																		
	CL	-0.247	-0.184	-0.202	-0.099	0.174	0.402**	1																	
	PN	-0.057	-0.044	-0.231	-0.006	0.137	0.174	0.119	1																
	SPP	-0.055	-0.121	-0.021	0.021	0.195	0.056	0.346*	-0.252	1															
	PSS	-0.074	-0.433**	-0.234	0.197	0.053	0.104	0.466**	-0.024	0.076	1														
Cold water irrigated plot	*DTH*	-0.152	-0.097	0.216	-0.073	0.145	0.974**	0.425**	0.179	0.089	0.111	1													
	CL	-0.241	-0.167	-0.078	-0.099	0.237	0.397**	0.831**	0.173	0.234	0.501**	0.421**	1												
	PN	-0.201	-0.099	-0.129	-0.107	0.065	0.142	0.057	0.263	-0.25	0.257	0.132	0.131	1											
	SPP	0.333	0.007	0.293	0.272	0.071	0.25	-0.045	-0.111	0.297*	0.008	0.294*	-0.029	-0.156	1										
	PSS	-0.020*	-0.257	-0.192	0.142	-0.043	0.216	0.418**	0.186	0.297*	0.403**	0.226	0.487**	0.093	0.027	1									
	LDS	-0.375*	0.023	0.07	-0.341*	-0.22	-0.089	0.395**	-0.1	0.327*	0.045	-0.023	0.348*	-0.054	-0.201	0.06	1								
	PE	0.017	0.032	-0.034	-0.009	0.218	-0.380**	-0.403**	-0.221	-0.099	-0.132	-0.388**	-0.503**	-0.028	0.066	-0.399**	-0.061	1							
	PAT	0.081	0.196	0.158	-0.049	-0.234	-0.406**	-0.356**	-0.131	-0.336*	-0.424**	-0.403**	-0.535**	-0.063	-0.344*	-0.539**	0.042	0.178	1						
	PAM	-0.007	0.257	-0.056	-0.169	0.159	-0.514**	-0.494**	-0.277**	-0.209	-0.27	-0.566**	-0.593**	-0.07	-0.12	-0.655**	-0.202	0.671**	0.430**	1					
Phenotype Differences between [illegible]	*D_DTH*	0.223	0.037	0.198	0.176	-0.025	-0.529**	-0.078	-0.035	0.122	0.016	-0.326*	-0.053	-0.104	0.067	0.001	0.300**	0.158	0.125	-0.001	1				
	R_CL	0.022	-0.013	-0.177	0.019	-0.106	-0.028	0.176	-0.115	0.171	-0.132	-0.038	-0.396**	-0.187	-0.016	-0.19	0.057	0.217	0.369**	0.225	-0.058	1			
	D_PN	-0.111	-0.041	0.095	-0.08	-0.066	-0.068	-0.073	-0.761**	0.068	0.195	-0.079	-0.074	0.425**	-0.001	-0.112	0.058	0.189	0.081	0.213	-0.038	-0.017	1		
	R_SPP	-0.279	-0.041	-0.242	-0.21	0.056	-0.117	0.311*	-0.127	0.557**	-0.014	-0.133	0.186	-0.095	-0.602**	0.169	0.430**	-0.14	0.07	-0.059	-0.006	0.195	0.055	1	
	R_PSS	0.016	0.206	0.141	-0.115	0.042	-0.225	-0.381**	-0.195	-0.309*	-0.228	-0.241	-0.439**	-0.032	-0.046	-0.979**	-0.071	0.392**	0.493**	0.665**	-0.008	0.163	0.162	-0.173	1
